# A natural small molecule isoginkgetin alleviates hypercholesterolemia and atherosclerosis by targeting ACLY

**DOI:** 10.7150/thno.105782

**Published:** 2025-03-18

**Authors:** Zhidan Zhang, Meijie Chen, Yitong Xu, Zhihua Wang, Zhenghong Liu, Chenyang He, Fanshun Zhang, Xiaojun Feng, Xiayun Ni, Yuanli Chen, Jixia Wang, Xinmiao Liang, Zhifu Xie, Jingya Li, Maciej Banach, Jaroslav Pelisek, Yuqing Huo, Yunhui Hu, Paul C Evans, Li Wang, Xiao-yu Tian, Jianbo Xiao, Yuhua Shang, Yijun Zheng, Xunde Xian, Jianping Weng, Suowen Xu

**Affiliations:** 1 Department of Endocrinology, Centre for Leading Medicine and Advanced Technologies of IHM, The First Affiliated Hospital of USTC, Division of Life Sciences and Medicine, University of Science and Technology of China, Hefei, 230001, China.; 2 Institute of Cardiovascular Sciences, State Key Laboratory of Vascular Homeostasis and Remodeling, School of Basic Medical Sciences, Peking University, Beijing, China.; 3 Key Laboratory of Metabolism and Regulation for Major Diseases of Anhui Higher Education Institutes, College of Food and Biological Engineering, Hefei University of Technology, Hefei, China.; 4 Key Laboratory of Separation Science for Analytical Chemistry, Dalian Institute of Chemical Physics, Chinese Academy of Sciences, Dalian 116023, China.; 5 State Key Laboratory of Drug Research, the National Center for Drug Screening, Shanghai Institute of Materia Medica, Chinese Academy of Sciences. Shanghai, 201203, China.; 6 Department of Preventive Cardiology and Lipidology, Medical University of Lodz 23 (MUL), Rzgowska 281/289, 93-338, Lodz, Poland.; 7 Department of Vascular Surgery, University Hospital Zurich, Zurich, Switzerland.; 8 Department of Ophthalmology, Baylor College of Medicine, Houston, Texas 77030, USA.; 9 Tasly Pharmaceutical Group Co., Ltd., Tianjin, 300410, China; National Key Laboratory of Chinese Medicine Modernization, Tianjin, 301617, China.; 10 Centre for Biochemical Pharmacology, William Harvey Research Institute, Barts and The London Faculty of Medicine and Dentistry, Queen Mary University of London, EC1M 6BQ, UK.; 11 Department of Biomedical Sciences, City University of Hong Kong, China.; 12 School of Biomedical Sciences, Chinese University of Hong Kong, NT, Hong Kong SAR, China.; 13 Universidade de Vigo, Department of Analytical and Food Chemistry, Faculty of Sciences, Ourense, 32004, Spain.; 14 Anhui Genebiol Biotech. Ltd., Hefei, 230000, China.; 15 Clinical Pharmacy (Sino-Foreign Cooperation) Class, School of Chinese Materia Medica, Tianjin University of Traditional Chinese Medicine, Tianjin 301617, China.; 16 Anhui Provincial Key Laboratory of Metabolic Health and Panvascular Diseases, Hefei, 230001, China.; 17 Institute of Endocrine and Metabolic Diseases, University of Science and Technology of China, Hefei, 230001, China.

**Keywords:** Atherosclerosis, Hyperlipidemia, ATP-Citrate lyase (ACLY), lipid metabolism, Isoginkgetin

## Abstract

**Rationale:** Atherosclerotic cardiovascular disease (ASCVD) represents the predominant cause of mortality and morbidity globally. Given the established role of hypercholesterolemia as a significant risk factor for ASCVD, the discovery of new lipid-lowering medications is of paramount importance. ATP citrate lyase (ACLY) is a crucial enzyme in cellular metabolism, providing acetyl-CoA as the building block for the biosynthesis of fatty acids and cholesterol. Consequently, it has emerged as a promising drug target for innovative treatments of lipid metabolic disorders.

**Methods:** Virtual screening of a natural product library was performed to identify small-molecule ACLY inhibitors, leading to the discovery of isoginkgetin (ISOGK). The lipid-lowering and anti-atherosclerotic effects of ISOGK were validated in hypercholesterolemic diet-induced animal models (mice and hamsters). The inhibitory effects of ISOGK on ACLY enzymatic activity were measured using commercial assay kits. The direct interaction between ISOGK and ACLY was confirmed by surface plasmon resonance (SPR) and cellular thermal shift assays (CETSA). Liver-specific ACLY knockdown mice were generated using GalNAc-conjugated siRNA (GalNAc-siAcly).

**Results:** ISOGK directly bind to ACLY and inhibit its enzymatic activity *in vitro* and *in vivo*. By inhibiting ACLY, ISOGK treatment thus alleviates hypercholesterolemia and atherosclerosis in mice and hamsters. However, ISOGK fails to attenuate lipid accumulation and the expression of lipid-metabolism related genes in Acly knockout or depleted hepatocytes. *In vivo*, the lipid-lowering and anti-atherosclerotic effects of ISOGK were reversed by hepatic knockdown of *Acly* via treatment with GalNAc-siAcly in mice.

**Conclusions:** Taken together, the present study identifies ISOGK as an effective and naturally-occurring small-molecule inhibitor of ACLY that limits hypercholesterolemia and atherosclerosis. ISOGK thus serves as a promising drug lead in cardiovascular therapeutics.

## Introduction

Atherosclerosis is a complex and multifaceted vascular disease characterized by the accumulation of lipids, inflammatory cells, and fibrous elements within the arterial walls 1-3. This pathological process can lead to the formation of atherosclerotic plaques, contributing to various cardiovascular diseases (CVDs), including stroke, peripheral vascular disease and coronary artery disease. The atherosclerotic cardiovascular disease (ASCVD) remains a major cause of morbidity and mortality worldwide 4.

An increasing body of research indicates that hypercholesterolemia has been identified as a significant risk factor for the development and progression of ASCVD 5-8. Statins, evident in their efficacy to reduce low-density lipoprotein cholesterol (LDL-C) levels and mitigate complications associated CVDs, have increased the accessibility of these drugs to patients 9, 10. Nevertheless, a significant number of high-risk patients fail to attain satisfactory reductions in LDL-C levels, experiencing intolerance to statins or are nonadherent to statin therapy mostly due to side effects related to the liver or skeletal muscles or new-onset diabetes 11, 12. Therefore, finding new medications to control blood lipids is crucial for treating atherosclerosis.

ACLY is a crucial enzyme in glucolipid metabolism that catalyzes the conversion of citric acid to acetyl-CoA, linking the tricarboxylic acid cycle (TCA) and lipid anabolism 13. The resulting cytosolic acetyl-CoA is a shared substrate for the synthesis of fatty acids and cholesterol 14, 15. Recent studies suggest that ACLY is an appealing target in cardiometabolic diseases ranging from hypercholesterolemia to metabolic liver diseases 16. Numerous inhibitors of ACLY have been discovered, of which bempedoic acid has attracted the most clinical attention. It is the first ACLY inhibitor to be approved by the FDA for patients resistant to statin therapy 17, 18. The recent cardiovascular outcomes phase 3 trial investigated the effect of bempedoic acid versus placebo on high and very high cardiovascular risk patients who are intolerant to statins and have high LDL-C levels (≥100 mg/dL) 19. Bempedoic acid led to a slight reduction in the primary composite endpoint for such individuals, highlighting its potential as an important treatment option for lipid disorders 19. Besides bempedoic acid, incorporating an enedioic acid structural component, 326E emerges as an another ACLY inhibitor. Its LDL-C lowering capability was observed to be on par with atorvastatin during a study on rhesus monkeys over two weeks, achieving reductions of 20.1% and 19.4% from the baseline, respectively 20. In a recent study, we have designed a liver-targeted siRNA against Acly (Aclysiran) by conjugating GalNAc and shown that Aclysiran attenuate hyperlipidemia and atherosclerosis comparable to Bempedoic acid 8. These studies indicate that ACLY inhibitors hold great potential for treating dyslipidemia.

Despite ongoing progress in rational drug design, natural products with validated ALCY inhibitory effects *in vivo* are lacking. *Ginkgo biloba* L. leaf extract (GBE), a crucial element in traditional Chinese medicine, has been employed clinically to address conditions such as chest arthralgia, heartache, stroke, and other ailments attributed to blood stasis 21, 22. Studies indicate that GBE can lower blood lipid levels in rats with metabolic dysfunction-associated steatotic liver disease (MASLD), though the specific bioactive components and target mechanisms involved remain unclear 23. Isoginkgetin (ISOGK), derived from *Ginkgo biloba*, belongs to the biflavonoid family and is a 3′,8″-dimerization product of its equivalent monoflavone residues 24. In this study, ISOGK was identified as a potential interactor with ACLY through virtual screening, and its capacity to inhibit ACLY enzyme activity was subsequently demonstrated *in vitro* and* in vivo*. Of translational impact, ISOGK exhibits robust lipid-lowering and anti-atherosclerotic effects in both mouse and hamster atheroprone models.

## Materials and Methods

### Animals

C57BL/6J (N000013, Gempharmatech, Nanjing, Jiangsu, China) and *Apoe*^-/-^ (T001458, Gempharmatech, Nanjing, Jiangsu, China) mice were employed. C57BL/6J mice had a standard chow diet for 4 weeks with concurrent treatment with ISOGK (HY-N2117, MedChemExpress, Piscataway, NJ, USA). Alipoprotein E knockout (*Apoe*^-/-^) mice were subjected to a 1.25% high cholesterol diet (#D12108C, RESEARCH DIETS, New Brunswick, NJ, USA). After 7 weeks of atherosclerosis induction, intraperitoneal injection of ISOGK (20 mg/kg/day) 25 was initiated and continued for additional 8 weeks. Upon reaching the designated experimental endpoint, blood was collected retro-orbitally, and serum was isolated for subsequent biochemical parameter analysis. Subsequently, the aorta and heart tissues were processed. The aorta was subjected to Oil Red O staining (#G1260, SolarBio, Beijing, China) for *en face* examination, and cryosections of the aortic sinus were prepared for analysis as we previously described 26. The serum lipid profile was evaluated utilizing colorimetric kits from Leidu Biotech (Shenzhen, China). Liver samples were gathered, processed into a homogenate, and then centrifuged. The resulting transparent supernatant was utilized for assessing hepatic triglyceride (TG) and total cholesterol (TC) concentrations using kits obtained from commercial sources (290-63701 for TG, 294-65801 for TC; Wako, Tokyo, Japan and E-BC-K261-M for TG, E-BC-K109-M for TC; Elabscience, Wuhan, China). The levels of TG and TC were normalized against the weight of the liver tissue. Kits for antioxidant-related indicators, including GSH (S0059S, Beyotime, Shanghai, China), MDA (S0131S, Beyotime, Shanghai, China), SOD (S0101S, Beyotime, Shanghai, China), and ox-LDL (CSB-E07933m, CUSABIO, Beijing, China), were detected in mouse serum. Acly^flox/flox^ mice were sourced from the Shanghai Model Organisms Center (no. NM-CKO-200076). The Institutional Animal Care and Use Committee of the University of Science and Technology of China (USTC) approved all animal care procedures (approval number USTCACUC27120124102, USTCACUC212401038).

Syrian golden hamsters were purchased from Vital River Laboratories (Beijing, China). Hamsters were fed a high cholesterol diet and received ISOGK (5 mg/kg/day) for 4 weeks. Before being sacrificed, liver and blood samples were collected at the end of the experiment. The animal study protocol was approved by the Ethics Committee of Hefei University of Technology (approval number HFUT20221031001) and conducted in accordance with the guidelines set forth in the "Guide for the Care and Use of Laboratory Animals," published by the National Institutes of Health (NIH).

Syrian golden hamsters with a deficiency in the low-density lipoprotein receptor (*Ldlr*) gene were generated using the clustered regularly interspaced short palindromic repeats (CRISPR) and CRISPR-associated protein 9 (Cas9) gene editing system, as previously described 27. Male *Ldlr^-/-^* hamsters at 8-week-old were randomly divided into three groups. The animals were administered with vehicle, ISOGK (2 mg/kg/day), or ISOGK (5 mg/kg/day) for a period of four weeks. The hamsters were maintained on a 14-h light/10-h dark cycle at 24 °C and were fed a 1.25% high cholesterol diet. Unless otherwise stated, the hamsters were fasted for 12 h prior to euthanasia. All experiments were conducted in accordance with the principles of experimental animal care (NIH publication no. 85-23, revised 1996) and were approved by the Animal Care and Use Committee of Peking University (approval number LA2022-147).

### Pharmacokinetics study

Pharmacokinetic properties were investigated using ICR mice weighing between 18 and 22 grams. Six mice were assigned into two groups, with three in each group. ISOGK was administered via intragastric and tail vein injections. For both the intravenous and intragastric administration groups, cheek blood samples of approximately 0.05 mL were collected at 5 min, 15 min, 30 min, 1 h, 2 h, 4 h, 6 h, 8 h, and 24 h post-administration. The plasma concentration of compound in the mice was measured using the LC-MS/MS technique. Pharmacokinetic parameters were then analyzed using WinNonlin software.

### Primary hepatocytes isolation

Primary hepatocytes were isolated from male C57BL/6J mice fed a high-fat diet (HFD, 60 kcal% fat, 20 kcal% protein, 20 kcal% carbohydrate; D12492; RESEARCH DIETS, New Brunswick, NJ, USA) for 16 weeks according to our previous study 28. At the end of experiment, the mice were initially anaesthetized and subsequently subjected to perfusion with Liver Perfusion Medium (17701-038; Thermo Fisher Scientific, Waltham, MA, USA) via the portal vein, followed by Liver Digestion Medium (17701-034, Thermo Fisher Scientific, Waltham, MA, USA). Following perfusion, the liver was extracted and passed through a 100 μm cell strainer. The resulting cells were subjected to centrifugation at 50×g for 1 min, after which cells were incubated in DMEM supplemented with 10% FBS (F05-001-B160216; Bio-One Biotechnology, Guangzhou, China) and 1% penicillin-streptomycin (15140-122; Gibco by Invitrogen, Carlsbad, CA, USA) at 37 °C.

### Patch clamp recording method

The whole-cell patch clamp technique was employed for the experiments. Previously published works detail the methodologies and solutions used for capturing various currents in cardiomyocytes and in HEK cell lines that stably express different K^+^ channels 29. Specifically, the standard bath solution with 5 mM K^+^ consisted of 135 mM NaCl, 5 mM KCl, 2 mM CaCl_2_, 1 mM MgCl_2_, 10 mM glucose, and 10 mM HEPES, adjusted to pH 7.4 using NaOH. Meanwhile, the bath solution containing 135 mM K^+^ was made of 135 mM KCl, 2 mM CaCl_2_, 1 mM MgCl_2_, 10 mM glucose, and 10 mM HEPES, with the pH set to 7.4 by adding KOH. All experiments using the patch clamp method were conducted at a controlled room temperature of 22 ± 1 °C.

### Analysis of lipid profile and distribution by FPLC

Plasma levels of TC, TG, LDL-C, and high-density lipoprotein cholesterol (HDL-C) were determined following the manufacturer's instructions. Liver tissues (30-40 mg) were homogenized in 0.5 mL of cold PBS for hepatic TG and cholesterol measurement. A 0.4 mL homogenate was combined with 1.4 mL of a CHCl_3_/CH_3_OH mixture (2:1, v/v) and shaken overnight at room temperature. Following centrifugation at 2,500 × g for a period of 10 min, the organic phase was meticulously transferred and permitted to air-dry. The residual organic material was then reconstituted in 800 μL of ethanol containing 1% Triton X-100, whereby the ethanol served to solubilize the material.

Fast protein liquid chromatography (FPLC) was employed to analyze plasma lipoproteins using 200 μL of pooled plasma samples. The samples were initially filtered through 0.22-µm filters and subsequently loaded onto Tricorn high-performance Superose S-6 10/300GL columns (Amersham Biosciences). Elution was conducted with PBS at a constant flow rate of 0.25 mL/min. The eluted fractions (500 μL each) were subjected to analysis for TG and TC concentrations using the aforementioned commercial assay kits.

### RNA extraction, reverse transcription, and RT-qPCR

Total RNA was extracted from cultured cells or animal tissue using RNeasy kits (RN001-50Rxns, YiShan, Shanghai, China). Subsequently, cDNA was generated using a Takara Reverse Transcription Kit (RR037B, Takara, Kyoto, Japan). Subsequently, quantitative real-time polymerase chain reaction (RT-qPCR) assays were conducted using ChamQ SYBR qPCR Master Mix (Q311-02, Vazyme, Nanjing, China), following the reverse transcription. The Roche LC96 real-time PCR detection system was employed for the detection of results, with glyceraldehyde-3-phosphate dehydrogenase (GAPDH) serving as the loading control for the quantification of mRNA levels. The fold change relative to the respective controls was determined using the 2^(^-ΔΔCt^) method. Primers were designed using the PrimerQuest software and subsequently synthesised by Sangon Biotech (Shanghai, China) 30. The sequences of primers used are provided in **Table S1**.

### Western blot

Western blotting was performed as previously described 31. In brief, whole-cell lysates from cultured cells and animal tissue were prepared using 1× sample buffer, followed by boiling at 95 °C for 10 min. Subsequently, SDS-PAGE was employed to separate the samples, which were then transferred onto a nitrocellulose membrane (Pall, NY, USA). Subsequently, the membrane was incubated in a blocking buffer for one h at room temperature. Following the removal of the blocking buffer, the blots were incubated with primary antibodies (1:1,000 dilution) at 4 °C overnight. The membrane was washed thrice for 10 min each, using 1× Tris-buffered saline with 0.1% Tween-20 (TBST). Subsequently, the membranes were incubated with IRDye® 680RD Goat anti-Mouse IgG (H + L) or IRDye® 800CW Goat anti-Rabbit IgG (H + L) (1:10,000 dilution, LI-COR, Lincoln, Nebraska, USA) at room temperature for 1 h. Visualization of the blots was carried out using the Li-COR CLx infrared imaging system 26. GAPDH (60004-1-Ig, Proteintech, Chicago, IL, USA), ACLY (67166-1-Ig, Proteintech, Chicago, IL, USA), LDLR (10785-1-AP, Proteintech, Chicago, IL, USA), β-Actin (66009-1-Ig, Proteintech, Chicago, IL, USA), AMPK (10929-2-AP, Proteintech, Chicago, IL, USA), p-AMPK^T172^ (2535, Cell Signaling Technology, Danvers, MA, USA).

### ELISA

Cytokines, including C-reactive protein (CRP) (RK04303, ABclonal, Guangzhou, China), interleukin-1 beta (IL-1 β) (RK00006, ABclonal, Guangzhou, China), and interleukin-6 (IL-6) (RK00008, ABclonal, Guangzhou, China), were extracted from mouse serum and analyzed using enzyme-linked immunosorbent assay (ELISA) kits. All procedures were conducted in accordance with the instructions provided by the manufacturers.

### Nile red staining

For the Nile Red staining procedure, primary mouse hepatocytes were treated with IOSGK at varying concentrations for a period of 12 h. Subsequently, the cells were fixed with 4% paraformaldehyde (PFA) and stained with Nile Red (N1142, Invitrogen, Carlsbad, CA, USA). The intracellular accumulation of lipids was visualized and quantified using a laser scanning confocal microscope (TCS SP8; Leica, Wetzlar, Germany) 28.

### Measurement of *de novo* lipogenesis *in vitro*

Hepatocytes were seeded in 6-well plates at a density of 6 x 10^5^ cells per well. They were exposed to various compounds along with [1,2-^14^C]-acetate (0.1 μCi per well) for specified durations. Following treatment, the cells underwent washing with cold PBS and were lysed in 0.4 to 0.6 mL of 0.5 M KOH. The saponification process at 95 °C for 3 h with 0.4 mL of KOH (in 20% methanol solution) enabled the separation of cholesterol and fatty acids. Cholesterol, being nonpolar, was extracted using 0.5 mL of petroleum ether in three separate instances. In comparison, polar fatty acids were extracted with 0.5 mL of petroleum ether after adding 0.2 mL of H_2_O and 0.4 mL of 5 M H_2_SO_4_ in three rounds. The petroleum ether extracts were left to air dry at room temperature overnight. The radioactivity of the resultant products was quantified using a liquid scintillation counter 20.

### ACLY activity assays *in vitro*

ACLY activity was assessed in a cell-free system using an ADP-Glo dependent assay, as previously described 32. Briefly, the reaction was conducted in a 384-well plate containing 2 µL of substrate (5 mmol/L ATP, 30 mmol/L citrate, 15 mmol/L CoA) in kinase buffer (40 mmol/L Tris base, 10 mmol/L MgCl_2_, 5 mmol/L DTT). The reaction was initiated by adding 20 nmol/L recombinant human ACLY protein and various concentrations of the compounds, followed by incubation for 30 min at 37 °C. During the reaction, ACLY converted ATP to ADP. ADP levels, which reflect ACLY activity, were quantified using the ADP-Glo assay kit (V9102, Promega, Madison, USA) in accordance with the manufacturer's instructions. The enzymatic activity curve was analyzed using the log (inhibitor) versus normalized response-variable slope function in GraphPad Prism.

### Measurement of ACLY activity* in vivo*

The activity of the ACLY enzyme was assayed utilizing malate dehydrogenase (MDH)-coupled method kits (BC4240, Solarbio, Beijing, China). In summary, liver extracts were incubated in a reaction buffer comprising 20 mM citrate, 10 mM MgCl₂, 10 mM DTT, 0.5 U/ml of malic dehydrogenase, 0.33 mM CoASH, and 0.14 mM NADH, both in the presence and absence of 5 mM ATP. The quantity of oxaloacetate produced by ACLY catalysis was determined by measuring the alteration in absorbance at 340 nm, which corresponds to the depletion of NADH during the MDH-catalyzed reaction. After accounting for background variations in absorbance in the absence of ATP, the relative ACL activities were computed by normalizing the data to the weight of the liver tissue.

### Molecular docking analysis

The crystal structure of ACLY (Protein Data Bank ID: 6O0H) was retrieved from the protein database and underwent energy minimization in preparation for use as the receptor structure for molecular docking studies. The substrates were constructed and hydrogenated, and then underwent structural optimization using the molecular orbital package program. Molecular docking was conducted using the Autodock Vina 1.2.0 software, with the docking box encompassing the active site and a docking time of 100 33. All other parameters were maintained at their default settings.

### Surface plasmon resonance

The interaction between isoginkgetin and ACLY was examined using an OpenSPR instrument (Nicaya, Canada). The recombinant ACLY protein was immobilized on a carboxyl sensor chip (SEN-AU-100-3-COOH; Nicaya, Canada) via a standard amine coupling method. Isoginkgetin was injected into the running buffer at various concentrations (ranging from 0.5 to 8 μM) at a flow rate of 20 μL/min. The interaction parameter KD was determined using TraceDrawer evaluation software based on the 1:1 Langmuir binding model.

### DiI-LDL uptake assay

HepG2 cells were cultured in 24-well plates and incubated in DMEM supplemented with 2% LPDS and 20 µg/mL DiI-LDL (20614ES76, YEASEN, Shanghai, China) for four h at 37 °C in the dark. Subsequently, the cells were rinsed twice with PBS containing 0.4% albumin and then washed three times with PBS. To quantify the fluorescence, 400 µL of isopropanol was added to each well, and the plates were incubated at room temperature for 20 min with continuous shaking. Subsequently, 200 µL aliquots were analyzed using a SpectraMax M2e Microplate Reader (Molecular Devices) at wavelengths of 520-570 nm, as previously described previously 34.

### RNA sequencing and bioinformatic analysis

Total RNA was extracted, and cDNA libraries were generated to investigate differences in gene expression. The Illumina sequencing platform was employed for single-end sequencing of the libraries. HISAT2 software (version 2.0.5) was utilized to align the reads to the mouse reference genome in Ensembl. StringTie (version 1.3.3b) was employed to compute raw counts of genes. DESeq2 (version 1.20.0) software was used to normalize the count matrix. Gene sets were established based on each KEGG pathway term and the associated genes, with GSEA conducted using the Java GSEA platform (version 3.0). This analysis utilized the 'Signal2Noise' metric for ranking and adopted a permutation type specific to 'gene sets'. Gene sets displaying FDR values below 0.25 were deemed to hold statistical significance. The expression patterns of pivotal genes within the pathways enriched by GSEA were visually represented through heatmaps, generated by employing the "pheatmap" package within the R programming environment. The relevant data can be accessed via the Gene Expression Omnibus database, using the following accession number: GSE261858.

### LDL oxidation and agarose gel electrophoresis

Prior to oxidation, EDTA was removed from LDL solutions (YB-001; Yiyuan Biotechnologies, Guangzhou, China). Subsequently, LDL solutions at a concentration of 0.2 mg/mL were incubated with varying concentrations of ISOGK in PBS for one h at 37 °C. The oxidation process was initiated by combining both native LDL and LDL pretreated with ISOGK with copper sulfate (99.0% purity; Jinshanting New Chemical Reagent Factory, Shanghai, China) to achieve a final copper concentration of 5 μM in PBS. This mixture was then incubated at 37 °C. Agarose gel electrophoresis was performed utilizing 0.5% agarose gels prepared in a sodium barbital buffer, following the stipulated electrophoretic methodology. The procedure was carried out in a 0.075 M sodium barbital buffer at a constant voltage of 55 V for a duration of one h. The relative electrophoretic mobility (REM) was determined by calculating the ratio of the migration distance of oxidized LDL to that of native LDL.

### CCK8 assay

The cellular viability was assessed by conducting CCK8 assays in primary hepatocytes from C57BL/6J mouse and HepG2 cell line. Specifically, 2,000 cells per well were plated in 96-well plates and cultured with different concentrations of ISOGK for 12 h. Following this, cell absorption was quantified using a CCK8 kit (CA1210, Solarbio, Beijing, China), adhering strictly to the manufacturer's guidelines. The presented data represents the findings from three independent experiments, with each experiment consisting of four replicates.

### Cellular thermal shift assay

The AML12 cell lysates were separated into two portions; one served as a control, while the other was exposed to 5 µM ISOGK for 1 h at room temperature. Subsequently, the lysates were individually heated at the specified temperatures (50 to 90 °C) for 5 min and allowed to cool at room temperature. Following this, the lysates underwent centrifugation at 12,000 rpm for 10 min at 4 °C, after which the supernatants were analyzed through Western blot.

### Immunofluorescence

Tissue slides from the male *Apoe^-/-^* mice aortic sinus was washed with PBS and then permeabilized in a solution of PBS with 0.3% BSA and 0.03% Triton X-100 for 1 h at room temperature. These sections were subsequently incubated overnight with primary antibodies at a 1:400 dilution, followed by a 1-h incubation with Alexa Fluor 488 or 546-conjugated sary antibodies at a 1:2000 dilution, also at room temperature. After three rinses with PBS, the slides were mounted with an antifade medium that included DAPI. Fluorescent images were captured using a Leica TCS SP8 X confocal laser scanning microscope. Primary antibodies, CD68 (MCA1957, BIO-RAD, Hercules, CA, USA), α-SMA (14395-1-AP, Proteintech, Chicago, IL, USA), CD31 (28083-1-AP, Proteintech, Chicago, IL, USA). Sary antibodies, Alexa Fluor™ 488 (A-11006; Thermo Fisher Scientific, Waltham, MA, USA), Alexa Fluor™ 546 (A-11035; Thermo Fisher Scientific, Waltham, MA, USA).

### Statistical analyses

The results are expressed as the mean ± standard error of the mean (SEM). Sample sizes for each experimental group were determined based on preliminary pilot studies and a comprehensive review of the pertinent literature. A normality test was conducted to verify whether the sample data originated from a normally distributed population. For comparisons between two groups, paired t-tests were employed, while one-way or two-way analysis of variance (ANOVA) was utilized for comparisons across multiple groups, with Bonferroni correction applied to address multiple comparisons. Data analysis was performed using GraphPad Prism 8.0, and p-values less than 0.05 were considered statistically significant.

## Results

### Discovery of Isoginkgetin as a novel naturally-occurring ACLY inhibitor

**Figure 1:**
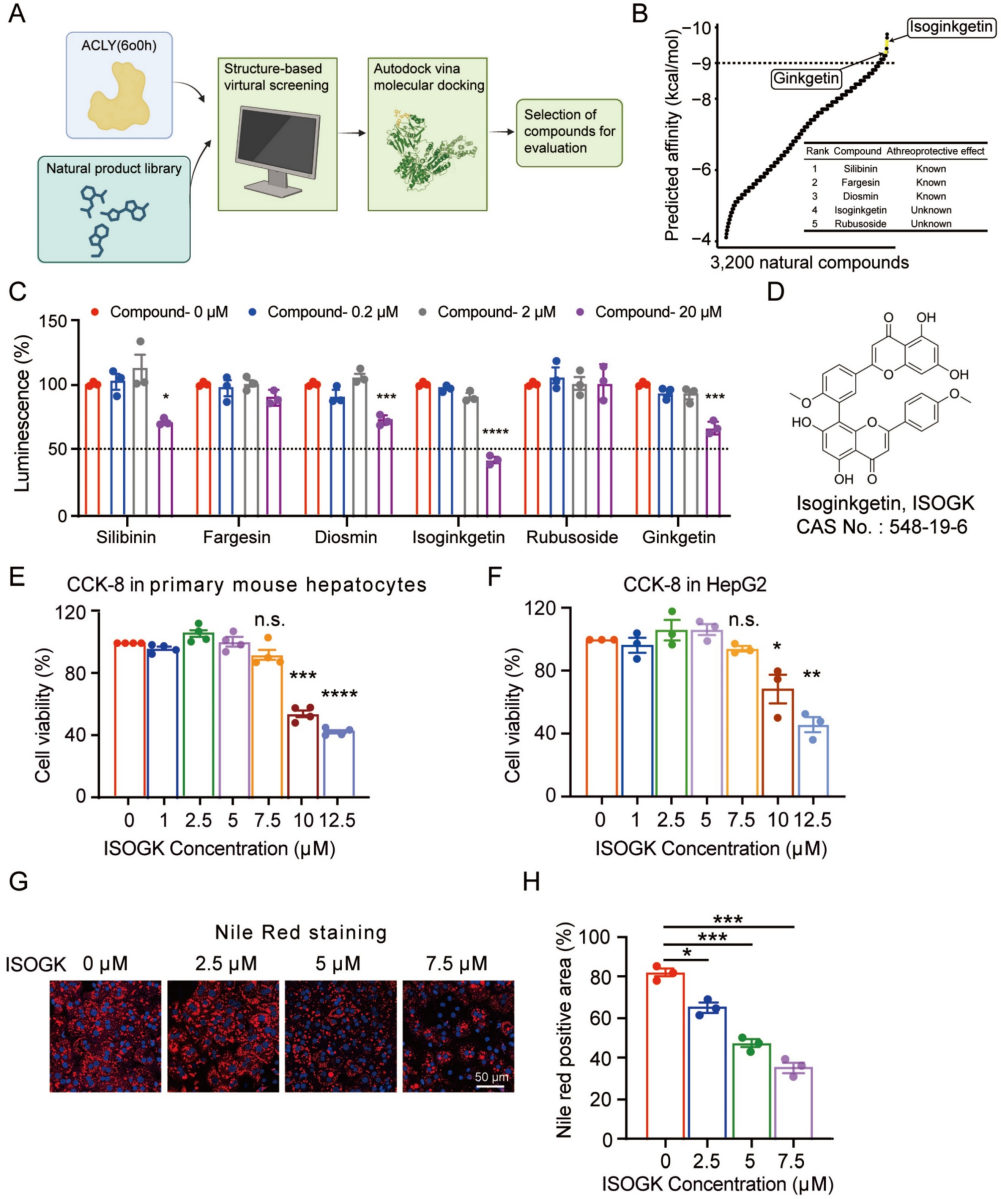
** Discovery of Isoginkgetin as a naturally-occurring ACLY inhibitor.** (A) Schematic diagram of molecular autodocking. Briefly, we conducted virtual molecular docking based on the structure of the ACLY protein to screen for potential natural small molecules that interact with ACLY from a natural product library comprising 3,200 compounds. (B) The predicted affinity results of A. (C) The inhibitory effect of the top candidates on ACLY enzyme activity was evaluated *in vitro* using the ADP-Glo assay (n = 3). One-way ANOVA followed by Bonferroni's post hoc test. Compared with compound 0 µM. (D) Chemical structure of ISOGK. (E) The cell viability in primary hepatocytes from C57BL/6J mouse treated with dose-dependent ISOGK detected by CCK-8 assay (n = 4). One-way ANOVA followed by Bonferroni's post hoc test. Compared with ISOGK 0 µM. (F) The cell viability in HepG2 cell line treated with dose-dependent ISOGK detected by CCK-8 assay (n = 3, technical replicates). One-way ANOVA followed by Bonferroni's post hoc test. Compared with ISOGK 0 µM. (G) Representative images of Nile Red staining in primary hepatocytes isolated from mice fed a high fat diet for 16 weeks which are treated with vehicle or indicated concentrations ISOGK for 12 h. (H) Quantitative analysis of G (n = 3). One-way ANOVA followed by Bonferroni's post hoc test. The data are means ± SEM, n.s., not significant, *P < 0.05, **P < 0.01, ***P < 0.001, ****P < 0.0001.

In order to identify novel ACLY inhibitors, we utilised virtual molecular docking to screen a natural compound library consisting of 3,200 natural products with the aim to identify novel ACLY inhibitor (**Figure 1A**). As illustrated in **Figure 1B**, the compounds are ranked according to their binding affinity in ascending order. The top three compounds, namely silibinin, fargesin and diosmin, have been demonstrated to exhibit anti-atherosclerotic properties 35-37. In addition, in the top 20 compound list, 4 compounds are derived from *Ginkgo biloba*, including ISOGK, bilobetin, sciadopitysin, and ginkgetin (**Table S2**). Based on the ranking score of identified compounds as well as criteria of unreported atheroprotective effects and the inhibition effect of ACLY activity measured by ADP-Glo assay kits *in vitro*, ISOGK was selected for further investigation as a potential natural ACLY inhibitor (**Figure 1C**).

ISOGK is a biflavonoid compound chemically defined as two apigenin units linked by a 3',8'' carbon-carbon bond, as shown in **Figure 1D**. CCK-8 result indicated that ISOGK exhibited no significant cytotoxicity in primary mouse hepatocytes and HepG2 cell line at concentrations below 7.5 μmol/L (**Figure 1E-F**), allowing us to select a safe dosage for downstream experiments. Primary hepatocytes from mice fed a 16-week high fat diet were then isolated and treated with varying concentrations of ISOGK. Nile Red staining results showed that ISOGK treatment reduced hepatic lipid levels (**Figure 1G-H**).

These data suggest that ISOGK functions as a natural inhibitor of ACLY and exerts lipid-lowering effects *in vitro*.

### Pharmacokinetic parameters and safety profile of isoginkgetin* in vivo*

We then investigated the pharmacokinetic properties of ISOGK in ICR mice through intravenous (5 mg/kg) and oral (20 mg/kg) administration. Following intravenous injection, the drug concentration in plasma reached its peak at 5 min, with a peak concentration of 710 ng/ml, and then gradually declined over time. However, the oral route of administration resulted in almost undetectable levels of ISOGK, suggesting low oral bioavailability of ISOGK (**Figure S1A**). Further, to evaluate the safety of ISOGK in normal mice, 8-week-old male C57BL/6J mice were administered 20 mg/kg/day by intraperitoneal injections (*i.p.*) (**Figure S1B**). Following a four-week period, fasting blood glucose (FBG) and body weight were evaluated, revealing no statistically significant differences between the vehicle- and ISOGK-treated groups prior to the mice being sacrificed for further analysis (**Figure S1C**). Meanwhile, the ratio of liver weight to body weight in the mice also showed no significant change. We also collected serum from the mice and assessed organ function-related index, such as alanine aminotransferase (ALT), aspartate aminotransferase (AST) and alkaline phosphatase (ALP) for liver function, blood urea nitrogen (BUN) and creatinine (CREA) for kidney function, and creatine kinase (CK) and creatine kinase-MB (CK-MB) for heart function. The administration of ISOGK did not induce any significant difference in these indicators when compared to the vehicle group (**Figure S1D**). Furthermore, the indicators reflecting organ damage, lactate dehydrogenase (LDH), also showed no variation (**Figure S1D**). Next, the major organs of mice, such as the heart, liver, spleen, lungs, and kidneys, underwent hematoxylin and eosin (H&E) staining. The results showed no histological differences between the two groups (**Figure S1E**). Additionally, periodic acid-schiff (PAS) staining of kidney tissues revealed no significant difference in glycogen deposits between both groups (**Figure S1F**).

To assess the potential cardiac safety of ISOGK, we used a manual patch clamp technique to examine its effect on HEK cells with stably expressed *hERG* channel currents. The *hERG* gene encodes for the pore-forming component of a critical potassium channel in cardiac tissue, which regulates potassium ion outflow in a membrane potential-dependent, gated manner. The results suggest that ISOGK had weak or no inhibitory effect on *hERG* channels, as the IC_50_ was greater than 30 μM (**Figure S1G**). These findings suggest that ISOGK is a safe compound when administered *in vivo* at tested doses.

### Isoginkgetin attenuates Western-diet induced-atherosclerosis in male and female *Apoe^-/-^* mice

**Figure 2:**
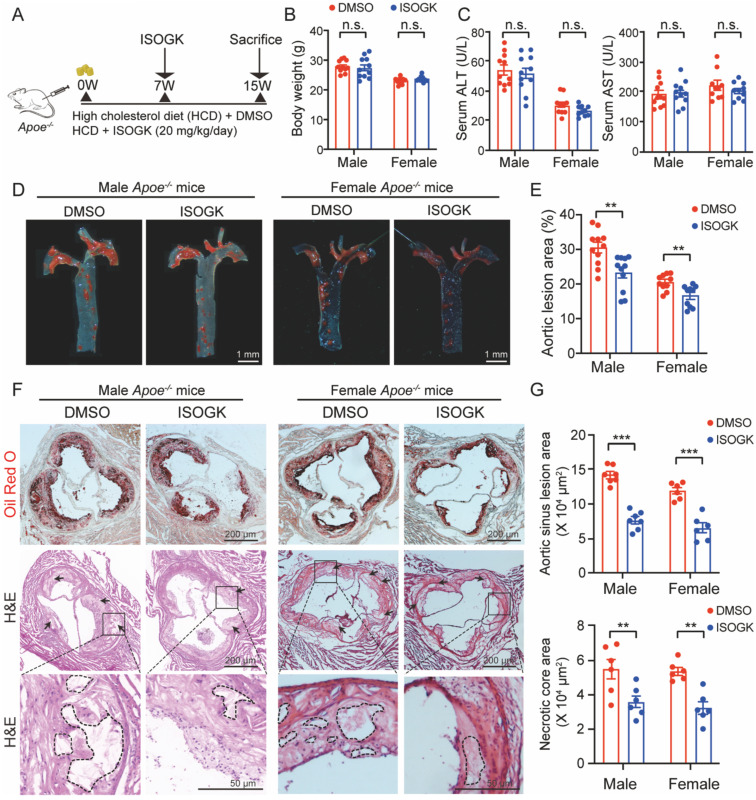
** Isoginkgetin attenuates atherosclerosis in male and female *Apoe^-/-^* mice.** (A) Scheme of study design. Male and female *Apoe^-/-^* mice were induced by high cholesterol diet for 7 weeks. Animals then received ISOGK treatment (20 mg/kg/day) for another 8 weeks. (B) The body weights were shown after ISOGK treatment for 8 weeks (n = 10 or 11). Two-tailed Student's t test. (C) Serum ALT, AST levels of *Apoe^-/-^* mice treated with or without ISOGK (n = 10 or 11). Two-tailed Student's t test. (D) Representative images of Oil Red O staining of *en face* aortas from male and female *Apoe^-/-^* mice fed high cholesterol diet. (E) Quantification of *en face* plaque areas as a percentage of the area in D (n = 10 or 11). Two-tailed Student's t test. (F) The representative images of Oil Red O (upper) staining and H&E (lower) staining of the aortic sinus from male and female *Apoe^-/-^* mice fed high cholesterol diet. Arrow indicates necrotic core area. (G) Quantitative analysis of F (n = 6 or 7). Two-tailed Student's t test. The data are means ± SEM, n.s., not significant, **P < 0.01, ***P < 0.001.

To ascertain the effects of ISOGK on atherosclerosis, we administered ISOGK via *i.p.* injection into both male and female *Apoe*^-/-^ mice fed a high cholesterol diet at the seventh week (**Figure 2A**). No significant changes in body weight were observed in either male or female mice in comparison to the vehicle group (**Figure 2B**). Furthermore, the serum levels of ALT and AST indicated that there were no significant changes in either male or female mice (**Figure 2C**). The H&E staining and Sirius red staining images also show that the livers of the ISOGK-treated mice exhibited no substantial pathological alterations (**Figure S2A-B**).

Administration of ISOGK at a dosage of 20 mg/kg/day 25 for 8 weeks resulted in a reduction of *en face* plaque areas in the aorta of *Apoe*^-/-^ mice, as observed by Oil Red O (ORO) staining. The lesion area was found to be significantly reduced in male (by 7.21%) and female (by 3.88%) mice treated with ISOGK in comparison to those treated with vehicle (**Figure 2D-E**). The analysis of ORO staining in the aortic sinus revealed a significant decrease in lesion area, by 46.35% and 45.74% in the ISOGK-treated group compared to the vehicle group in male and female *Apoe*^-/-^ mice (**Figure 2F-G**). Additionally, the arrows indicate the presence of a necrotic core in the aortic sinus, as observed through the use of H&E staining. The results demonstrate a notable reduction in the necrotic core area in the ISOGK treatment group (**Figure 2F-G**).

Furthermore, immunofluorescence assay was utilized to evaluate the alterations in the content of macrophages, smooth muscle cells, and endothelial cells within the aortic sinus of* Apoe*^-/-^ mice. The findings demonstrated that following the administration of ISOGK, the content of macrophages in the aortic sinus exhibited a substantial decline, while the content of smooth muscle cells and endothelial cells remained unaltered (**Figure S2C-D**). Serum inflammatory marker analysis was conducted using ELISA, encompassing C-reactive protein (CRP), interleukin 1 beta (IL-1β), and interleukin 6 (IL-6). The findings revealed no statistically significant differences in either male or female mice treated with vehicle or ISOGK (**Figure S2E-F**). These results indicated that ISOGK provides protection against atherosclerosis in *Apoe*^-/-^ mice without impacting systemic inflammation.

### Isoginkgetin modulates lipid metabolism in *Apoe^-/-^* mouse without impacting oxidative stress

**Figure 3:**
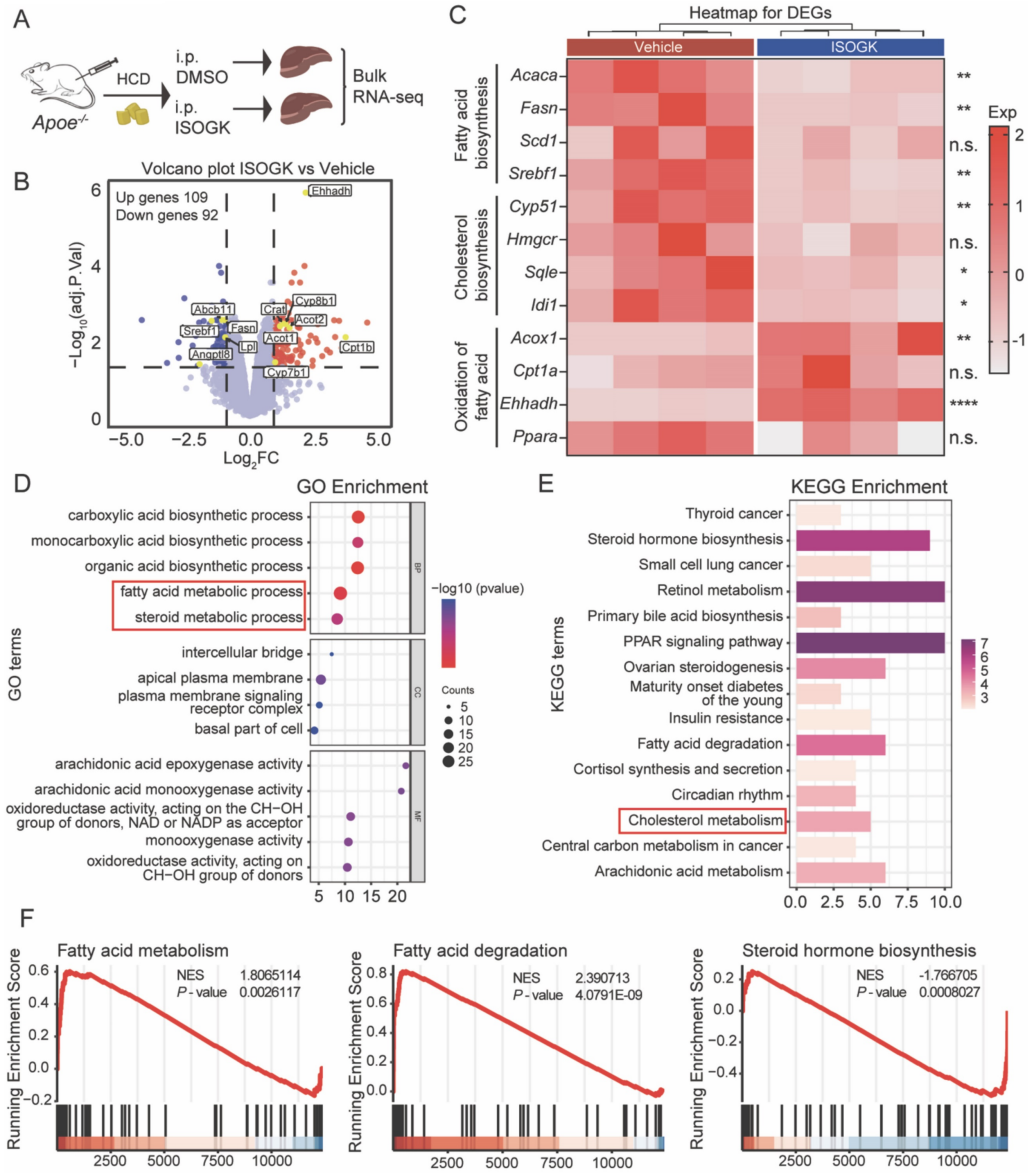
** Isoginkgetin modulates lipid metabolism in *Apoe^-/-^* mouse liver.** (A) Scheme of sample processing. (B) Volcano plot showing differential expression genes (adjusted P < 0.05, | log2 (Fold change) | > 1). (C) Heat map displaying the indicated gene expression in liver tissues from *Apoe^-/-^* mice with or without ISOGK (n = 4). (D) GO enrichment analysis of transcriptomes from *Apoe^-/-^* mice liver samples. (E) KEGG pathway enrichment analysis of transcriptomes from *Apoe^-/-^* mice liver samples. (F) GSEA analysis of transcriptomes from *Apoe^-/-^* mice liver samples. The data are means ± SEM, n.s., not significant, *P < 0.05, **P < 0.01, ****P < 0.0001.

To understand the effects of ISOGK on liver lipid metabolism, we conducted RNA sequencing on liver samples from vehicle and ISOGK-treatment groups of *Apoe*^-/-^ mice (**Figure 3A**). Comparative RNA-seq analysis in vehicle- and ISOGK-treated mouse liver showed differential expression of 201 genes, with 109 upregulated and 92 downregulated genes (adjusted p < 0.05, | log2 (Fold change) | > 1) (**Figure 3B**). Heat map analysis revealed that, subsequent to ISOGK treatment, the genes associated with fatty acid and cholesterol biosynthesis, including *Srebf1, Fasn, Acaca* and *Cyp51*, were significantly downregulated, while certain genes linked to fatty acid oxidation were substantially upregulated (**Figure 3C**). Next, the differentially expressed genes were enriched through Gene Ontology (GO) and Kyoto Encyclopedia of Genes and Genomes (KEGG) enrichment analyses. As depicted in **Figure 3D**, the GO enrichment analysis revealed alterations in the fatty acid metabolic process and steroid metabolic process. Additionally, the KEGG pathway analysis indicated a high enrichment of cholesterol metabolism (**Figure 3E**). The ISOGK treatment group significantly increased fatty acid degradation and fatty acid metabolism while inhibiting sterol biosynthesis, congruent with the role of ISOGK in ACLY inhibition (**Figure 3F**).

Given the notable antioxidant properties of biflavonoids 38, we extended our investigation to examine the antioxidant potential of ISOGK. Agarose gel electrophoresis was employed to evaluate its effects on LDL oxidation. The results indicated that, in comparison to the positive control group treated with Vitamin E, ISOGK at varying concentrations demonstrated no antioxidant activity against copper-induced LDL oxidation (**Figure S3A-B**). Concurrently, we assessed markers associated with oxidative stress in the serum of *Apoe^-/-^* mice. The findings revealed no significant alterations in these indicators (**Figure S3C-D**).

These data illustrate that ISOGK may alleviate atherosclerosis by regulating lipid metabolism without impacting oxidative stress.

### Isoginkgetin displays lipid-lowering effects in hyperlipidemia mice and hamsters

**Figure 4:**
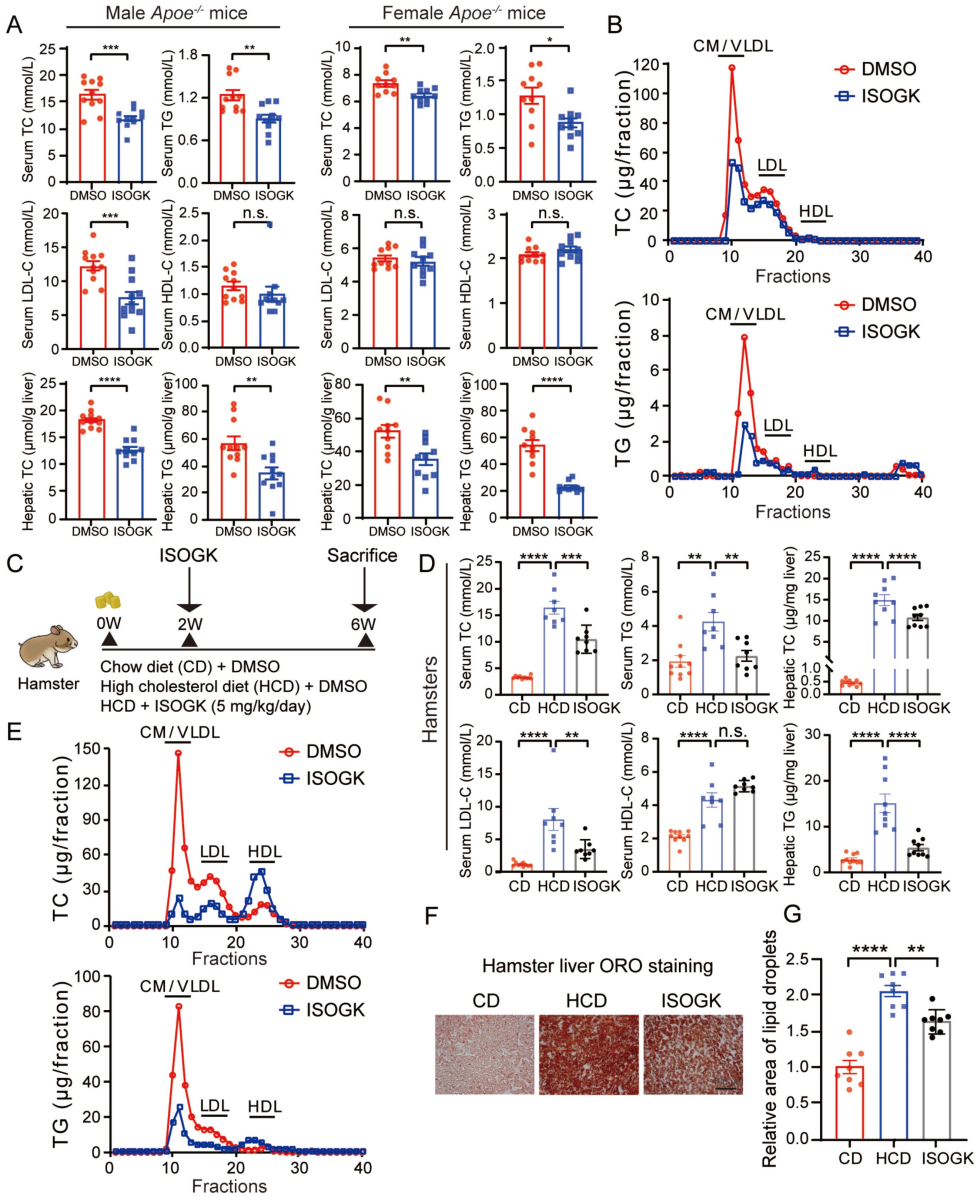
** Isoginkgetin displays lipid-lowering effects in hyperlipidemia mice and hamsters.** (A) Serum levels of TC, TG, LDL-C, HDL-C, and hepatic levels of TC, TG from male and female *Apoe^-/-^* mice fed high cholesterol diet 7 weeks and administrated ISOGK 8 weeks (n = 10 or 11). Two-tailed Student's t test. (B) The distribution of TC and TG in pooled plasma samples from the indicated female *Apoe*^-/-^ mice. (C) Study design of the hamster's experiment. (D) Plasma levels of TC, TG, LDL-C, HDL-C, and hepatic levels of TC, TG in the indicated hamster's group after treatment ISOGK or vehicle for 4 weeks (n = 8 - 10). One-way ANOVA followed by Bonferroni's post hoc test. (E) The distribution of TC and TG in pooled plasma samples from the indicated hamsters. (F) Representative images of Oil Red O staining in liver sections from the indicated groups. (G) Quantitative analysis of F (n = 8). One-way ANOVA followed by Bonferroni's post hoc test. The data are means ± SEM, n.s., not significant, *P < 0.05, **P < 0.01, ***P < 0.001, ****P < 0.0001.

To ascertain whether ISOGK regulates lipid metabolism, the plasma and hepatic lipid profiles of *Apoe*^-/-^ mice were evaluated. As shown in **Figure 4A**, ISOGK treatment resulted in a statistically significant decrease in TC levels in both male and female mouse serum (by 27.15% and 24.24%, respectively) and liver tissues (by 29.98% and 32.34%, respectively). Similarly, a notable decline in TG levels was observed in both serum and liver tissue, irrespective of gender. Furthermore, ISOGK decreased the serum levels of LDL-C in male mice and the levels of LDL-C and HDL-C in serum remained unchanged in female. To further determine which type of cholesterol is reduced in female mice, we used lipoprotein distribution analysis by FPLC. The results showed a significant reduction in the contents of TG and cholesterol carried in very low-density lipoprotein (VLDL) particles in the ISOGK-treated group (**Figure 4B**).

As Syrian golden hamster models offer advantages over traditional mouse models in lipid research due to their closer alignment with human physiology and higher sensitivity to diet-induced lipid abnormalities 39. Next, we conducted experiments using hamsters. At eight weeks of age, the hamsters were fed a high cholesterol diet for two weeks before commencing intraperitoneal ISOGK administration. Then animals were sacrificed after four weeks of ISOGK treatment (**Figure 4C**). The high cholesterol diet-fed hamsters treated with ISOGK had similar body weight compared to the vehicle-treated hamsters, and although their serum ALT and AST levels were higher than those of chow diet-fed hamsters, there was no significant difference between the ISOGK-treated and vehicle-treated groups (**Figure S4A-B**). The treatment duration of four weeks resulted in significant reductions in serum levels of TC, TG, and LDL-C (by 36.12%, 46.87% and 56.55%, respectively), as well as a significant decrease in hepatic lipid levels (TC lower by 27.53% and TG lower by 64.14%) in the ISOGK-treated group (**Figure 4D**). Additionally, FPLC analysis confirmed a significant decrease in serum TC and TG distribution in the ISOGK-treated hamsters (**Figure 4E**). Treatment with ISOGK also significantly reduced lipid deposition in hamster liver (**Figure 4F-G**). The serum antioxidant indicators were also detected, and the results demonstrated that they remained unaltered (**Figure S4C**).

These findings suggest that ISOGK has significant lipid-lowering effects in hyperlipidemic mice and hamsters.

### Isoginkgetin mitigates high cholesterol diet-induced hyperlipidemia and atherosclerosis in Ldlr^-/-^ hamsters

**Figure 5:**
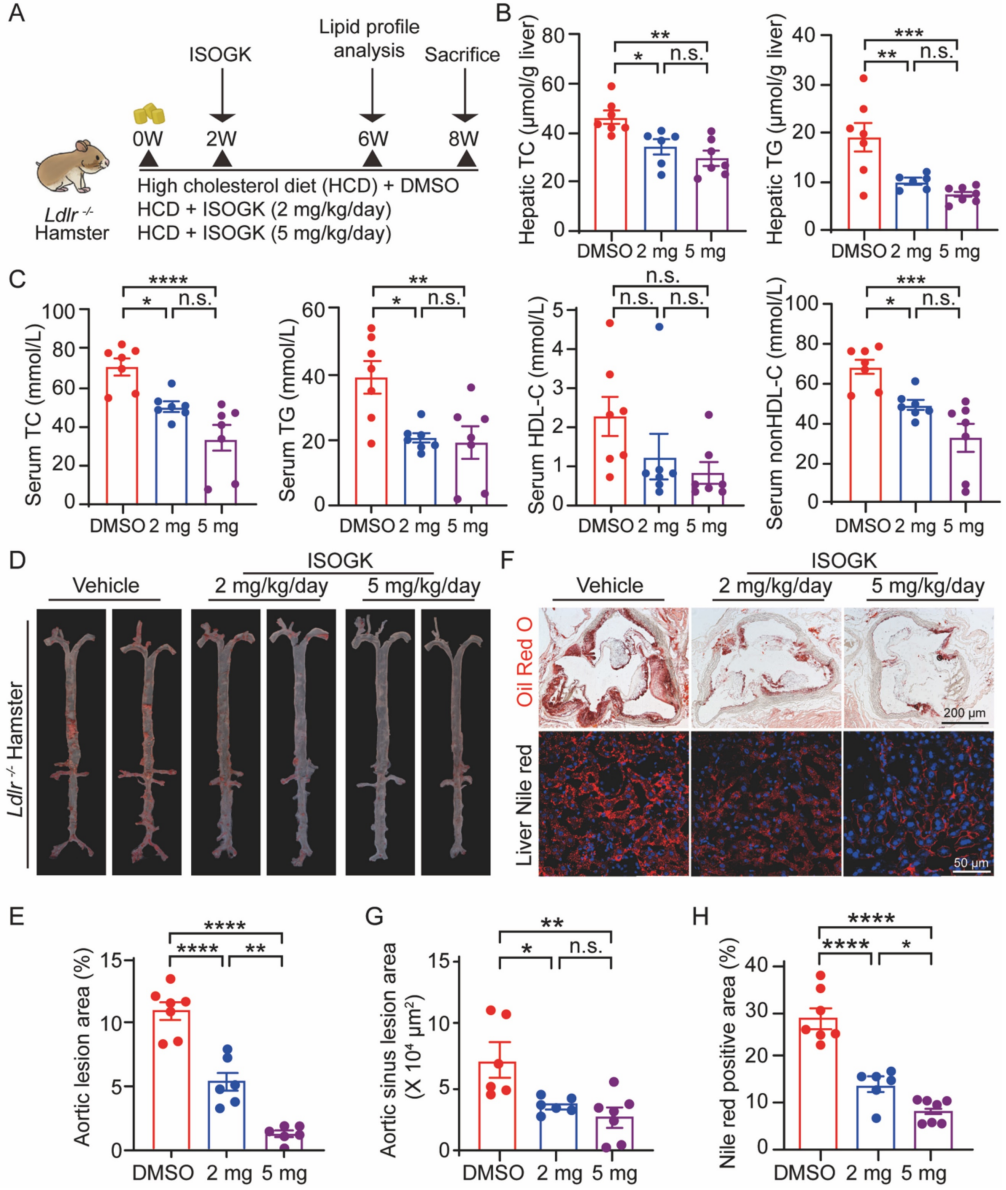
**Isoginkgetin ameliorates hyperlipidemia and atherosclerosis in *Ldlr^-/-^* hamsters.** (A) Scheme of study design. Briefly, 8-week-old male *Ldlr^-/-^* hamsters were induced by high cholesterol diet for 2 weeks and then administrated ISOGK (2 mg/kg/day and 5 mg/kg/day) treatment for another 6 weeks. (B) Hepatic levels of TC, TG in the indicated* Ldlr^-/-^* hamster's group after treatment ISOGK or vehicle for 8 weeks (n = 6 or 7). One-way ANOVA followed by Bonferroni's post hoc test. (C) Serum TC, TG, HDL-C and non-HDL-C levels in the indicated *Ldlr^-/-^* hamster's group after treatment ISOGK or vehicle for 6 weeks (n = 7). One-way ANOVA followed by Bonferroni's post hoc test. (D) Representative images of Oil Red O staining of *en face* aortas in indicated *Ldlr^-/-^* hamsters group. (E) Quantitative analysis of D (n = 6 or 7). One-way ANOVA followed by Bonferroni's post hoc test. (F) Representative images of Oil Red O staining in *Ldlr^-/-^* hamsters' aortic sinus and representative images of Nile Red staining in *Ldlr^-/-^* hamsters' liver tissues. (G-H) Quantitative analysis of F (n = 6 or 7). One-way ANOVA followed by Bonferroni's post hoc test. The data are means ± SEM, n.s., not significant, *P < 0.05, **P < 0.01, ***P < 0.001, ****P < 0.0001.

It is notable that homozygous low-density lipoprotein receptor-deficient (*Ldlr*^-/-^) hamsters exhibit hypercholesterolemia, hypertriglyceridemia and atherosclerosis similar to human 39, 40. To further investigate the therapeutic effects of ISOGK, we used 8-week-old male *Ldlr*^-/-^ hamsters. After the induction with hyperlipidemia for two weeks, intraperitoneal injections of ISOGK were administered for a period of six weeks. The hamsters were randomly assigned to three groups: a vehicle group, a low-dose ISOGK group (2 mg/kg/day), and a high-dose ISOGK group (5 mg/kg/day). At the conclusion of the treatment period, the hamsters were euthanized (**Figure 5A**). In comparison to vehicle-treated high cholesterol diet-fed *Ldlr^-/-^* hamsters, those treated with 2 or 5 mg/kg ISOGK exhibited a reduction in hepatic TC levels of 25.89% and 35.75%, respectively. Furthermore, ISOGK treatment resulted in a significant reduction in serum TG levels, with a 47.57% and 61.32% decrease observed in low and high dose of ISOGK-treated hamsters (**Figure 5B**).

Similarly, analysis of the serum lipid profile revealed that low and high doses of ISOGK treatment significantly reduced TC (by 28.72% and 52.15%) and TG (by 47.99% and 51.38%) levels, without affecting HDL-C levels. However, non-HDL-C levels were significantly decreased (by 28.16% and 51.76%; **Figure 5C**). It further demonstrated that ISOGK has a significant effect on lowering lipid levels.

Furthermore, Oil Red O staining of the aortas demonstrated that ISOGK treatment significantly reduced plaque area by 21.74 % (low-dose) and 29.85% (high-dose) compared to the vehicle group (**Figure 5D-E**). Likewise, Oil Red O staining of the aortic sinus and Nile Red staining of the liver tissues both showed that ISOGK significantly reduced lipid accumulation in *Ldlr*^-/-^ hamsters (**Figure 5F-H**).

These results provide further evidence for the lipid-lowering and anti-atherosclerotic effects of ISOGK in a more clinically relevant rodent model of hyperlipidemia and atherosclerosis.

### Isoginkgetin treatment improves hyperlipidemia via inhibiting ACLY activity

**Figure 6:**
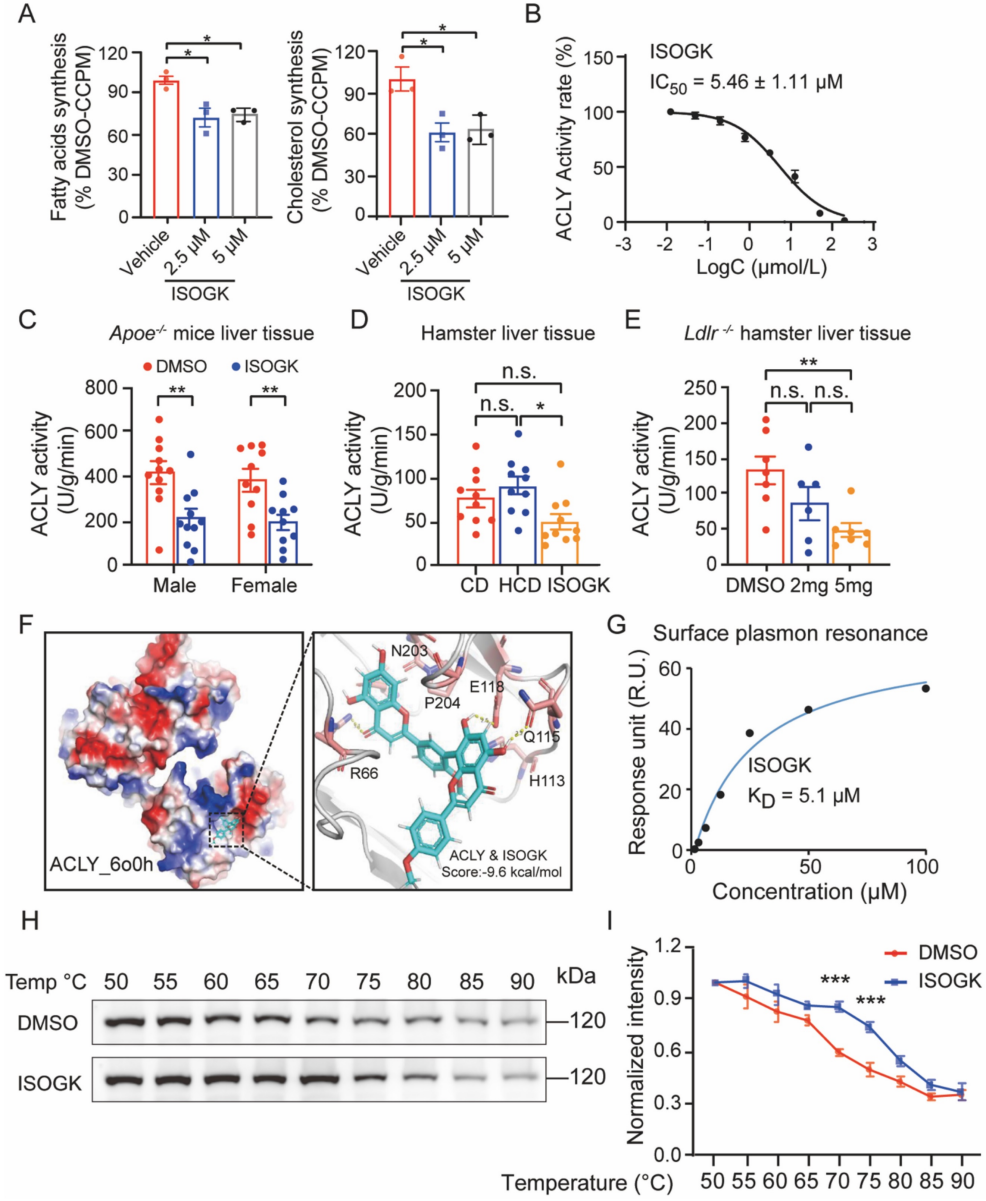
** Isoginkgetin inhibits ACLY activity *in vitro* and *in vivo.*** (A) Primary hepatocytes from C57BL/6J mice were cultured with vehicle or 5 µM ISOGK for 4 h in the presence of [1,2-^14^C]-acetate. The hepatic lipogenesis was calculated (n = 3). One-way ANOVA followed by Bonferroni's post hoc test. (B) Dose-dependent inhibition of purified, recombinant ACLY after incubation with ISOGK, the activity at control was defined as 100%. (C) ACLY enzyme activities were measured from the liver extracts of male and female *Apoe^-/-^* mice fed high cholesterol diet 7 weeks and administrated ISOGK 8 weeks (n = 10 or 11). Two-tailed Student's t test. (D). ACLY enzyme activities were measured from the liver extracts of hamster after treatment ISOGK or vehicle for 4 weeks (n = 10). One-way ANOVA followed by Bonferroni's post hoc test. (E) ACLY enzyme activities were measured from the liver extracts of *Ldlr^-/-^* hamster after treatment with or without ISOGK for 8 weeks (n = 6 or 7). One-way ANOVA followed by Bonferroni's post hoc test. (F) Molecular docking has predicted the binding sites of ISOGK with ACLY. (G) The interaction of ACLY with ISOGK was measured by surface plasmon resonance. (H) CETSA analyzed the thermal stabilization of ACLY with isoginkgetin in AML12 cell lysates. (I) Quantitative analysis of H (n = 3). Two-way ANOVA with Dunnett's T3 post hoc analysis. The data are means ± SEM, n.s., not significant, *P < 0.05, **P < 0.01, ***P < 0.001.

To evaluate the impact of ISOGK on *de novo* lipogenesis, primary mouse hepatocytes were incubated with ISOGK for 4 h. The incorporation rates of [1,2-^14^C]-acetate into fatty acids and cholesterol were measured. The results showed the inhibition of lipogenesis in response to ISOGK treatment at 2.5 μmol/L and 5 μmol/L (**Figure 6A**). We also tested the effect of ISOGK on ACLY inhibition in a cell-free system using the ADP-Glo assay. The results showed that the ISOGK potently inhibited ACLY enzyme activity with an IC_50_ = 5.46 ± 1.11 μmol/L (**Figure 6B**). The results indicate that ISOGK has a significant inhibitory effect on the enzymatic activity of ACLY *in vitro*. Consistently, ISOGK treatment also significantly inhibits ACLY activity *in vivo* via measured enzymatic activity in the liver tissue of three hyperlipidemia model animals (**Figure 6C-E**). Next, the interaction mode between ISOGK and ACLY was investigated, with a focus on the crucial amino acid residues. Molecular docking analysis revealed the presence of R66, H113, Q115, E118, N203, and P204 in the binding pocket of ACLY (**Figure 6F**). Furthermore, the direct interaction of ISOGK with ACLY was validated by surface plasmon resonance (SPR), which demonstrated an equilibrium dissociation constant (K_D_) of 5.1 μmol/L (**Figure 6G**). The cellular thermal shift assay (CETSA) analysis also indicated that ISOGK stabilized the ACLY protein during temperature-induced denaturation (**Figure 6H-I**).

The uptake and excretion of cholesterol in the liver are also of great importance with regard to blood lipid levels 41. To further assess the potential of ISOGK to alter LDL uptake, a DiI-LDL uptake assay was conducted in HepG2 cells. The results demonstrated that ISOGK had no significant effect on LDL uptake, irrespective of dose or time (**Figure S5A-B**). There was no significant difference in the color of bile collected from the gallbladders in *Apoe*^-/-^ mice treated with or without ISOGK (**Figure S5C**). Additionally, we analyzed the expression of genes associated with cholesterol efflux in the liver and found no significant differences between the two groups (**Figure S5D**). These supports the ISOGK treatment did not enhance cholesterol efflux in the liver. Next, we analyzed the protein levels of LDLR and ACLY, which are essential proteins that regulate cholesterol uptake and synthesis in hepatocytes. The expression of LDLR and ACLY protein levels were examined using Western blot analysis in the liver tissues of *Apoe*^-/-^ mice. The results indicated no significant change in either LDLR or ACLY protein expression (**Figure S5E**). We also found no significant change in the LDLR and ACLY protein levels in the human hepatocyte cell line Huh-7 cultured *in vitro* under various concentrations of ISOGK treatment (**Figure S5F-G**). Similarly, in the hyperlipidemic hamster model, the LDLR and ACLY protein levels in the liver showed no significant difference (**Figure S5H-I**). According to the literature 42, inhibitors of ACLY have been demonstrated to activate AMPK. Therefore, we assess the protein expression of p-AMPK via Western blot. The results demonstrated that ISOGK also exhibited a comparable capacity to activate AMPK (**Figure S5J-K**).

### The anti-atherosclerotic and lipid-lowering effects of isoginkgetin in *Apoe^-/-^* mice are ACLY dependent

Male and female Acly^flox/flox^ mice were used to isolate PMH and infected with adenovirus vectors carrying either Cre or GFP for 24 h. Western blot verification confirmed that the Cre treatment group significantly reduced the expression of ACLY protein (**Figure S6A**). The application of Nile red staining revealed that ISOGK did not further diminish lipid deposition in PMH when ACLY was abrogated or depleted (**Figure S6B**). This finding suggests that ISOGK exerts its regulatory effects by impeding ACLY activity. Concurrently, in AML12 and Huh7 cells, it was observed that following ACLY knockdown, the ISOGK treatment group did not demonstrate a further reduction in the mRNA expression of lipid synthesis-related genes *Srebf1c* and *Fasn* (**Figure S6C-D**). Collectively, these *in vitro* experimental results provide compelling evidence to suggest that ACLY is the target of ISOGK.

**Figure 7:**
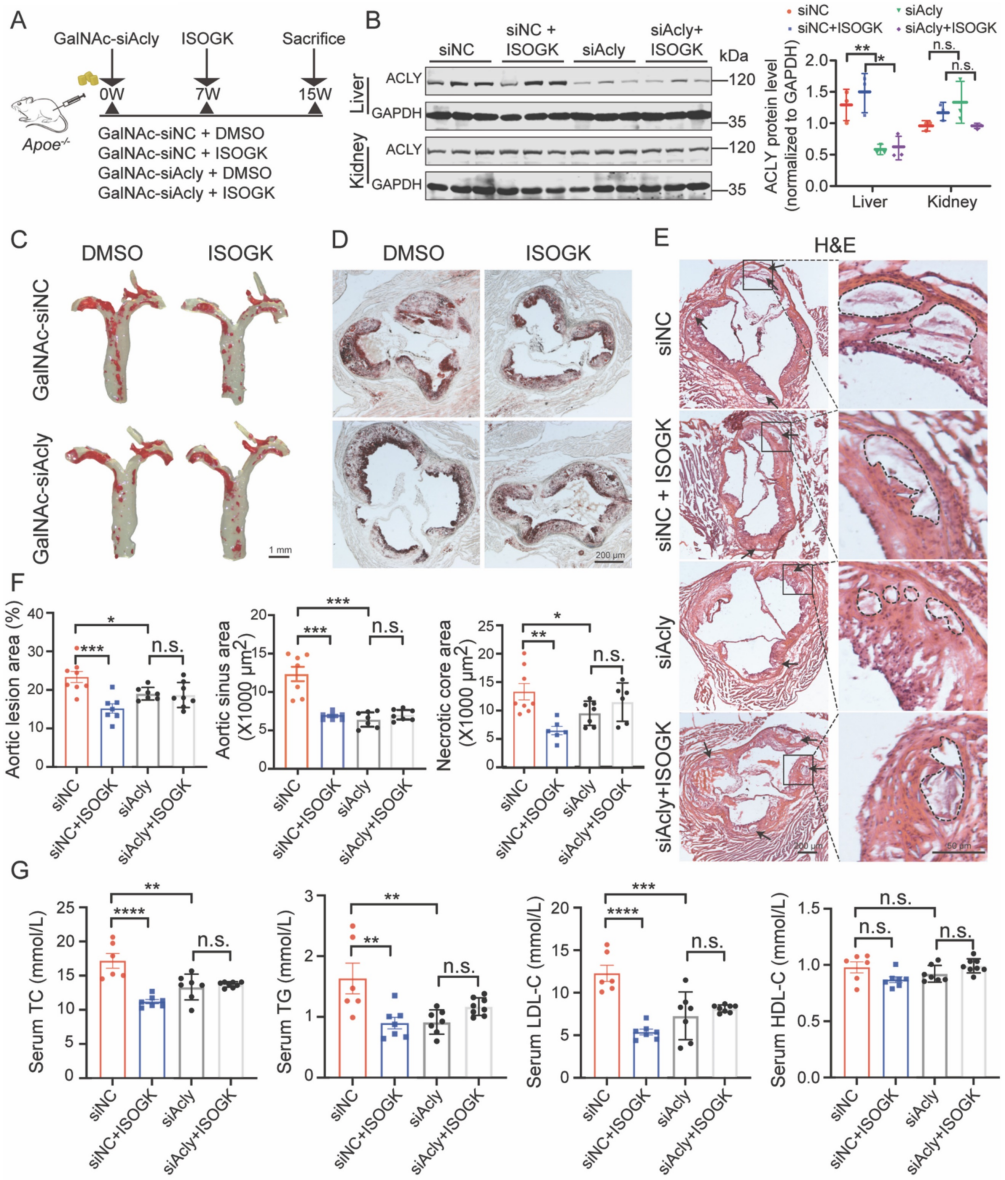
**The anti-atherosclerotic and lipid-lowering effects of isoginkgetin in *Apoe^-/-^* mice were ACLY-dependent.** (A) Scheme of study design. Briefly, male *Apoe^-/-^* mice were induced by high cholesterol diet for 7 weeks and administrated GalNAc-siAcly (3 mg/kg/month). Animals then received ISOGK (20 mg/kg/day) treatment for another 8 weeks. (B) Immunoblotting analysis and quantification of ACLY protein in liver and kidney tissues from indicated groups (n = 3). One-way ANOVA followed by Bonferroni's post hoc test. (C) Representative images of Oil Red O staining of *en face* aortas from male *Apoe^-/-^* mice fed high cholesterol diet. (D) Representative images of ORO staining of the aortic sinus from male *Apoe^-/-^* mice fed high cholesterol diet. (E) Representative images of H&E staining of the aortic sinus from male *Apoe^-/-^* mice fed High cholesterol diet. (F) Quantitative analysis of C-E (n = 6 - 8). One-way ANOVA followed by Bonferroni's post hoc test. (G) Plasma levels of TC, TG, LDL-C and HDL-C in the indicated groups (n = 6 - 8). One-way ANOVA followed by Bonferroni's post hoc test. The data are means ± SEM, n.s., not significant, *P < 0.05, **P < 0.01, ***P < 0.001, ****P < 0.0001.

Conjugating siRNA with N-acetylgalactosamine (GalNAc) is a promising strategy for depleting target gene expression specifically in the liver. GalNAc is a targeting ligand that exhibits high selectivity for asialoglycoprotein receptor 1 (ASGR1), which is abundantly and specifically expressed in hepatocytes 43, 44. In this study, we employed GalNAc-siAcly to knock down Acly in the liver of *Apoe*^-/-^ mice that were fed high cholesterol diet for 15 weeks. ISOGK was administered starting from the 7^th^ week for 8 additional weeks (**Figure 7A**). The protein level of ACLY in the mouse liver was significantly decreased in the GalNAc-siAcly group, and no apparent changes were detected in the kidneys by Western blot assay (**Figure 7B**). The qRT-PCR results also supported the same conclusion (**Figure S7A**). The body weight and blood glucose of the *Apoe*^-/-^ mice assessed at the time of euthanasia, and no significant differences were found among the indicated groups (**Figure S7B**). These results suggest successful and specific knockdown of *Acly* in the liver of *Apoe*^-/-^ mice using the GalNAc conjugation strategy.

To determine whether ISOGK mediated anti-atherosclerotic effects depends on ACLY inhibition, we analyzed the plaque area of the whole aorta and the aortic sinus in control mice and GalNAc-siAcly treated mice. The size of aortic plaques in ISOGK-treated *Apoe*^-/-^ mice was significantly lower compared to the vehicle group. Additionally, in the mice of the *Acly* knockdown group, the plaque area was not decreased further by ISOGK (**Figure 7C**). Analysis of Oil Red O staining and H&E staining in the aortic sinus revealed that the lesion areas and necrotic core area of the ISOGK-treated group were significantly reduced. While, the effect of ISOGK treatment on atherosclerosis was not observed in *Acly* knockdown* Apoe*^-/-^ mice (**Figure 7D-F**). Also, the lipid-lowering effect of ISOGK was not further observed in *Acly* knockdown *Apoe^-/-^* mice (**Figure 7G**). In conclusion, the results indicate that the anti-atherosclerotic and lipid-lowering effects of ISOGK depends on ACLY inhibition.

## Discussion

ASCVDs are the leading cause of global morbidity and mortality 45, 46. Due to unhealthy lifestyles and an aging population, the prevalence of ASCVDs is increasing 47, 48. Identifying potential drugs for the prevention and treatment of atherosclerosis is therefore a major medical need. LDL-C is causally related to the risk of atherosclerosis and its complications from evidence gleaned from preclinical and clinical studies 49, 50. In addition, Nordestgaard *et al*. 51 reported that individuals with familial hypercholesterolemia attain the LDL-C burden threshold at an earlier stage, resulting in the accelerated progression of atherosclerotic diseases. Lipid-lowering drugs, such as statins, PCSK9 inhibitors, ezetimibe and fibrates, have significantly contributed to the management of dyslipidemia 52. However, the use of first-line statins can be associated with some side effects, such as muscle pain, liver enzyme elevation, and increased risk of onset diabetes, which may somehow limit their utility 9; additionally, for some of the patients at high to extremely high CVD risk, more intensive LDL-C reduction may be necessary 53. In this regard, natural products are alternative or complementary therapies for managing hyperlipidemia 54.

*Ginkgo biloba* L., is an eminent herbal preparation derived from the dried leaves of ginkgo trees. The extract of *Ginkgo biloba* is widely used in the clinical prevention and treatment of various diseases. It is traditionally known for promoting blood circulation, activating meridians to alleviate pain, dissolving turbidity, and reducing lipid levels 55, 56. ISOGK, a biflavonoid derived from *Ginkgo Biloba*, is a known RNA splicing inhibitor 57. It has been reported to have various protective effects, including anti-inflammatory, antioxidant, and anti-tumorigenic properties 58, 59. In fibrosarcoma, ISOGK hindered the expression of MMP9 by disrupting NF-κB signaling, consequently impeding the invasion of HT1080 cells 60. ISOGK demonstrated pro-apoptotic effects in HeLa cells by enhancing the expression of BAX and cleaved Caspase 3 58. Additionally, ISOGK induced the unfolded protein response (UPR) and autophagy pathway by suppressing the activity of the 20S proteasome, resulting in cell death in Hela cells 61. It has also been identified as a promising pharmacological candidate for mitigating obesity-induced oxidative stress and cardiomyocyte damage via the activation of NRF2 62. Our findings in this experimental study demonstrate that ISOGK treatment did not induce any evident signs of toxicity in the liver, kidney, spleen, lung, or heart *in vivo*. Histological analyses showed no tissue damage or abnormalities in the organs. Additionally, we demonstrated that ISOGK does not significantly inhibit the *hERG* channel current, further supporting the potential cardiac safety of ISOGK. Our study on the effect of ISOGK treatment on high cholesterol diet-induced atherosclerosis in *Apoe*^-/-^ mice unravel a significant reduction in aortic plaques, regardless of gender. Furthermore, there was a reduction in both the plaque area and the necrotic core area in the aortic sinus. There were no significant changes in serum markers of inflammation, such as CRP, IL-1β, and IL-6. Further investigation is required to determine whether ISOGK possesses anti-inflammatory effects in the local atherogenic milieu.

Based on the liver RNA-sequencing results, it was discovered that ISOGK primarily regulates the metabolism of fatty acids and cholesterol. Overall, the findings suggest that ISOGK has potential therapeutic benefits for metabolic disorders. The study demonstrated that ISOGK treatment protects against atherosclerosis induced by high cholesterol diet in *Apoe*^-/-^ mice by lowering the LDL-C levels. Additionally, in the hamster model of diet-induced hypercholesterolemia, ISOGK has a significant lipid-lowering effect.

ISOGK has been demonstrated to possess antioxidant properties *in vivo* and to promote Nrf2 stability by hindering its proteosome-dependent degradation, thereby safeguarding against obesity-induced cardiomyopathy 62. However, the agarose gel electrophoresis experiment demonstrated that ISOGK did not effectively inhibit the oxidation of LDL in our study. The redox-related indicators GSH, MDA, SOD, and ox-LDL were measured in *Apoe*^-/-^ mice serum, and no significant difference were observed. Further experimental results *in vivo* are necessary to exclude the potential contribution of ISOGK to atheroprotection by preventing oxidative stress.

Statins have been the cornerstone of CVD management, but statin intolerance and residual risk have highlighted the need for novel targets with good safety profile, and low risk of nonadherence and discontinuation 63. ACLY is an essential enzyme in the cholesterol and fatty acid biosynthesis pathway. It converts citrate to acetyl-CoA, which is a precursor for cholesterol and fatty acid synthesis. Inhibiting ACLY has emerged as a promising approach to managing hyperlipidemia by reducing hepatic lipogenesis and TG-VLDL secretion 20, 64, 65. This reduction in hepatic *de novo* lipogenesis offers a distinct therapeutic mechanism compared to statin therapy. Our results show that ISOGK can inhibit the synthesis of fatty acids and cholesterol in primary mouse hepatocytes. Additionally, ISOGK significantly inhibits the activity of ACLY, as confirmed *in vitro* and *in vivo*. Knocking down ACLY in the liver of mice via GalNAc-siAcly abolished the therapeutic effects of ISOGK. Altogether, these results suggest that ISOGK lowers circulating and hepatic cholesterol levels by inhibiting ACLY activity.

When interpreting the data presented in this study, it is important to consider several limitations. Firstly, although the beneficial effect of ISOGK in the treatment of diet-induced atherosclerosis in mouse and hamster models has been demonstrated, the therapeutic efficacy of this agent in other models of atherosclerosis, such as those involving blood flow disturbance (disturbed blood flow induced by partial carotid ligation), remains uncertain. Furthermore, as a flavonoid compound, the metabolic form of ISOGK *in vivo* remains unclear, including whether it can be metabolized to monoflavonoid compounds. The pharmacophore of ISOGK that are responsible for its effects will continue to be subjected to analysis, and structural optimization may be necessary to improve its solubility and bioavailability. In addition, its safety and efficacy in large animal models of atherosclerosis are warranted in future studies. Further, to investigate the specific cellular effects of ISOGK within atherosclerotic lesions, it would be beneficial to use single-cell transcriptomics or spatial transcriptomics to provide detailed insights into the responses of various cell types (such as immune cells, vascular endothelial cells, and smooth muscle cells) to ISOGK treatment. Most important of all, clinical research is required to investigate the potential therapeutic benefits of ISOGK in the treatment of atherosclerosis in clinical settings.

## Conclusions

In summary, this study provides the first preclinical evidence in support of the lipid-lowering and atheroprotective of ISOGK by targeting ACLY enzyme activity. These findings indicate that the novel ACLY inhibitor ISOGK holds promise for clinical translation and suggest the potential therapeutic value of ISOGK in treating atherosclerotic vascular diseases.

## Supplementary Material

Supplementary figures and tables.
