# Supplementary material for: A natural small molecule isoginkgetin alleviates hypercholesterolemia and atherosclerosis by targeting ACLY
Source: Theranostics. 2025 Mar 18;15(10):4325–44. doi: 10.7150/thno.105782 (PMC11984407; doi:10.7150/thno.105782)
Supplement: Supplementary file 1 — Supplementary figures and tables. [file thnov15p4325s1.pdf]

Supplemental Materials for

A natural small molecule isoginkgetin alleviates hypercholesterolemia and atherosclerosis by targeting ACLY

Figure S1 to S7

Tables S1

Tables S2

Figure S1

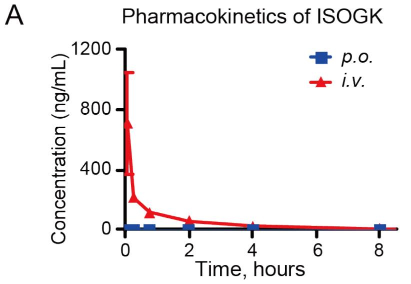

The pharmacokinetic parameters of ISOGK following *i.v.* administration in mice

Animal No.	$T_{1/2}$ (h)	AUC_{last} (h*ng/mL)	AUC_{INF_obs} (h*ng/mL)	$CL_{\_obs}$ (mL/min/kg)	MRT_{INF_obs} (h)	$Vss_{\_obs}$ (mL/kg)
1	1.75	415	433	192	2.73	31552
2	1.55	521	536	156	1.63	15208
3	0.85	338	348	239	0.821	11791
**Mean**	**1.38**	**424**	**439**	**196**	**1.73**	**19517**
**SD**	**0.47**	**92**	**94**	**42**	**0.96**	**10562**

Comment: The *p.o.* group has insufficient measured values to calculate pharmacokinetic parameters.

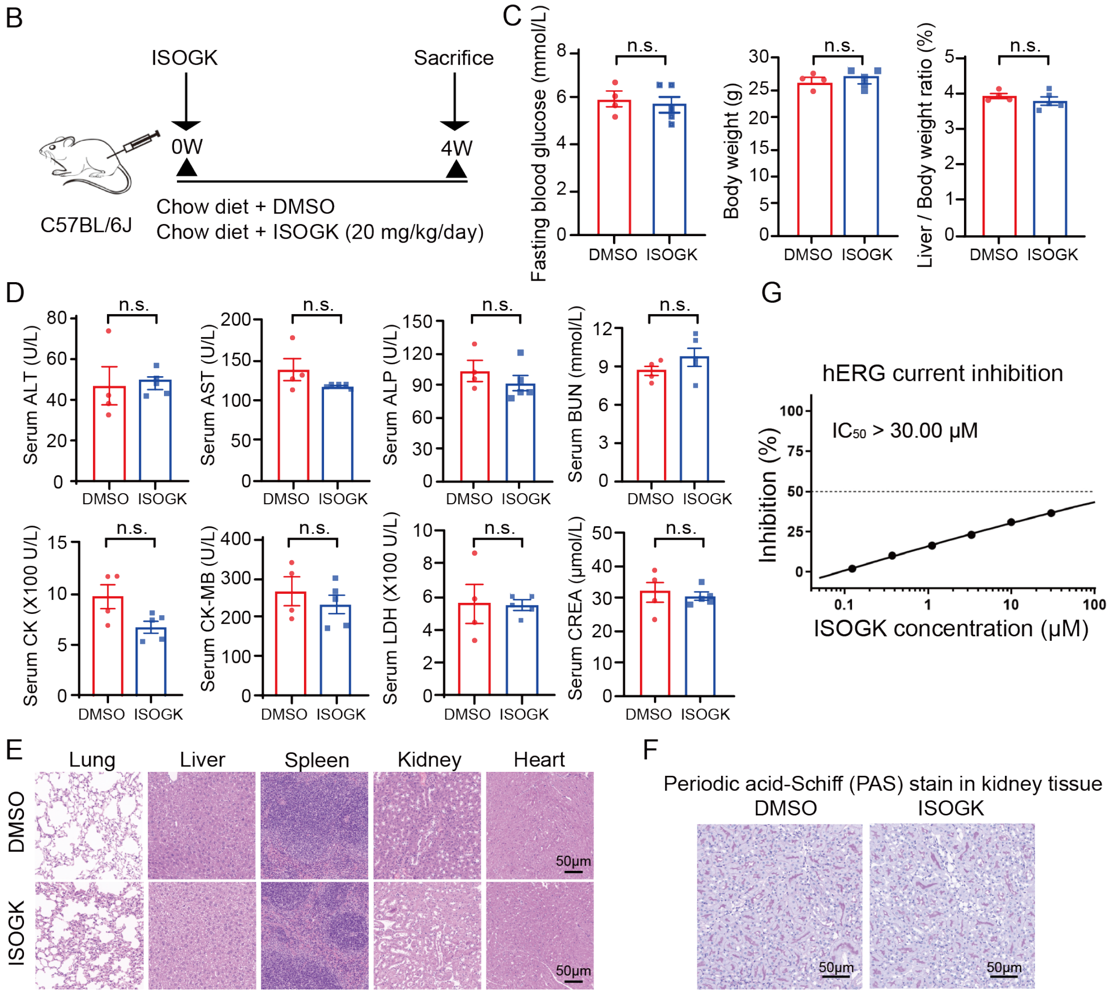

Fig. S1 Pharmacokinetic parameters and safety profile of Isoginkgetin *in vivo*

(A) Pharmacokinetics parameters of ISOGK after oral (*p.o.*) (20 mg/kg) or intravenous injections (*i.v.*) (5 mg/kg) administration in male ICR mice. (B) The protocol for safety assessment in male C57BL/6J mice administered 20 mg/kg/day ISOGK by intraperitoneal injections (*i.p.*). (C) The levels of blood glucose, body weight and liver-body weight ratio in C57BL/6J mice after treatment with or without ISOGK 4 weeks (n = 4 or 5). Two-tailed Student's t test. (D) Serum ALT, AST, ALP, BUN, CK-MB, CK,

LDH, and CREA levels of C57BL/6J mice treated with or without ISOGK (n = 4 or 5). Two-tailed Student's t test. (E) Represent H&E staining images of lung, liver, spleen, kidney, and heart tissues in C57BL/6J mice treated with vehicle or ISOGK. (F) Periodic acid-Schiff (PAS) staining images in the indicated mouse kidney tissue. (G) The inhibitory effect of ISOGK on hERG potassium channels. The data are means ± SEM, n.s., not significant.

Figure S2

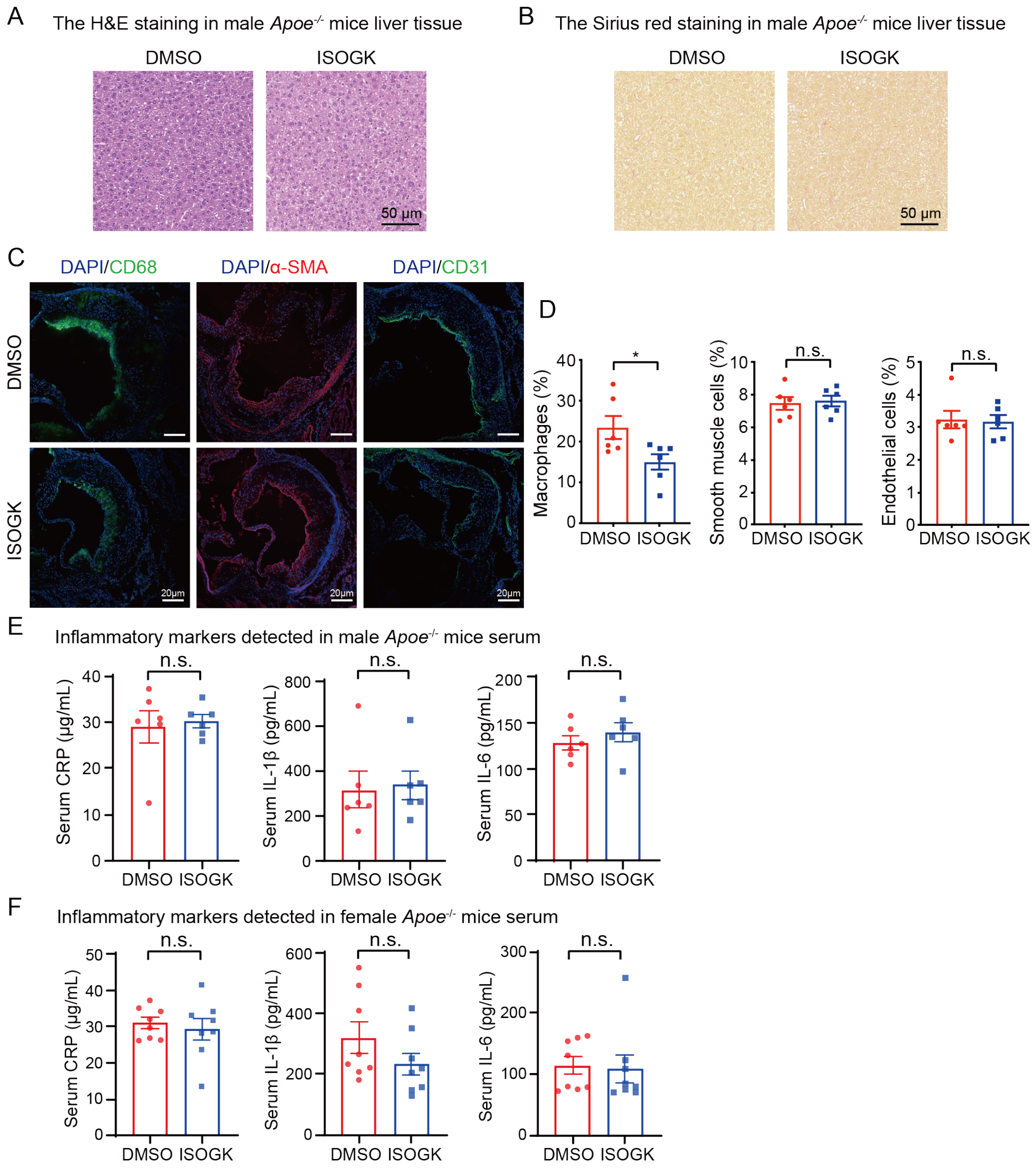

Fig. S2 Histopathological analysis and serum indicators in *Apoe*$^{-/-}$ mice treated with or without ISOGK

(A) Represent H&E staining images of liver tissues in male *Apoe*$^{-/-}$ mice treated with vehicle or ISOGK for 8 weeks. (B) Represent Sirius red staining images of liver tissues in male *Apoe*$^{-/-}$ mice treated with vehicle or ISOGK for 8 weeks. (C) CD68, and CD31 immunofluorescence staining in aortic sinus from vehicle- and ISOGK-treated male *Apoe*$^{-/-}$ mice. (D) Quantification of CD68, α-SMA and CD31 positive area (n = 6). Two-

tailed Student's t test. (E) Serum inflammatory cytokine CRP, IL-1β and IL-6 levels in male *Apoe*$^{-/-}$ mice treated with vehicle or ISOGK (n = 6). Two-tailed Student's t test. (F) Serum inflammatory cytokine CRP, IL-1β and IL-6 levels in female *Apoe*$^{-/-}$ mice treated with vehicle or ISOGK (n = 8). Two-tailed Student's t test. The data are means ± SEM, n.s., not significant. *$P < 0.05$.

Figure S3

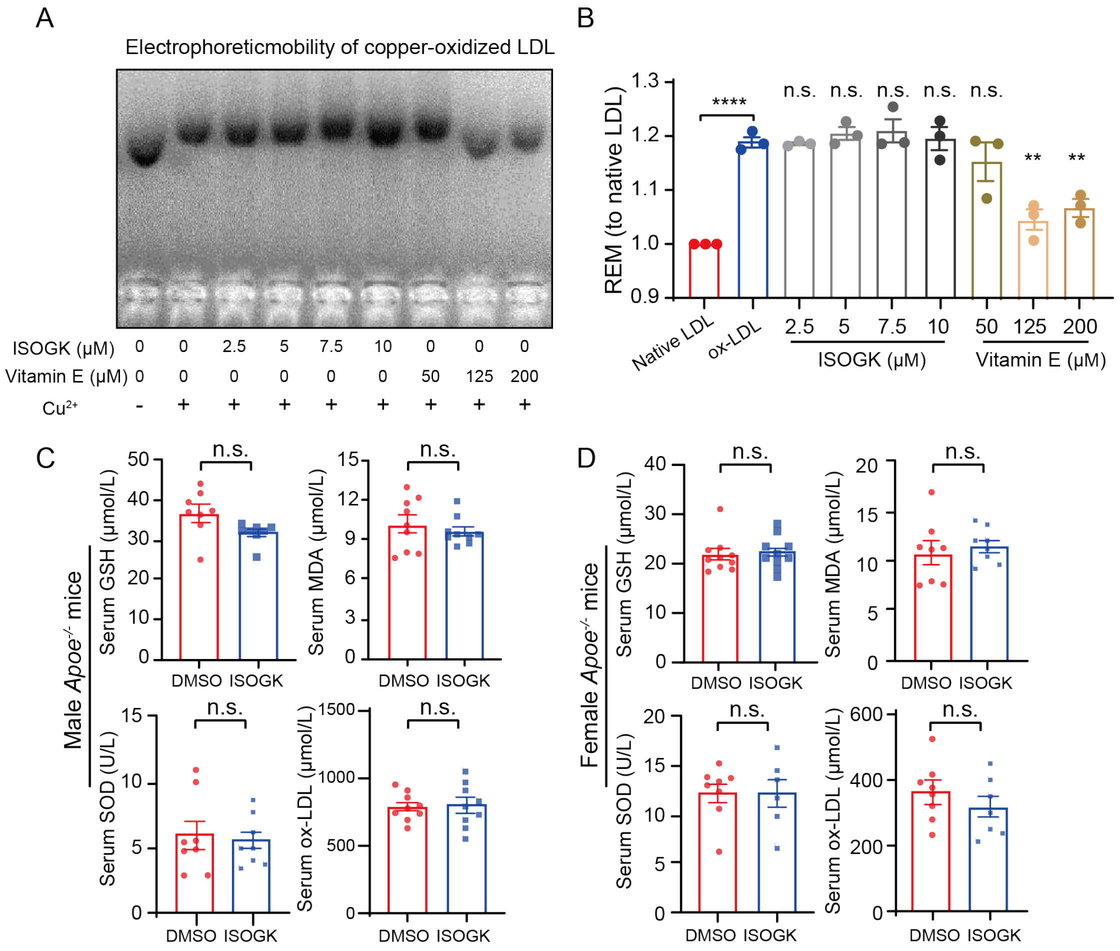

Fig. S3 Isoginkgetin does not affect oxidative stress.

(A) Agarose gel electrophoresis image of the LDL samples treated with or without ISOGK in the presence or absence of copper (5 μM) for 6 h. (B) Calculated relative electrophoretic mobility (REM) of native or oxidized LDL in the presence of indicated compounds (n = 3). Vitamin E treatment as the positive control which retards LDL oxidation. One-way ANOVA followed by Bonferroni's post hoc test. (C) Plasma GSH, MDA, SOD, ox-LDL levels from male *Apoe*$^{-/-}$ mice treated with vehicle or ISOGK for 8 weeks (n = 8 - 9). Two-tailed Student's t test. (D) Plasma GSH, MDA, SOD, ox-LDL levels from male *Apoe*$^{-/-}$ mice treated with vehicle or ISOGK for 8 weeks (n = 6 - 10). Two-tailed Student's t test. The data are means ± SEM, n.s., not significant. **$P < 0.01$, ****$P < 0.0001$.

Figure S4

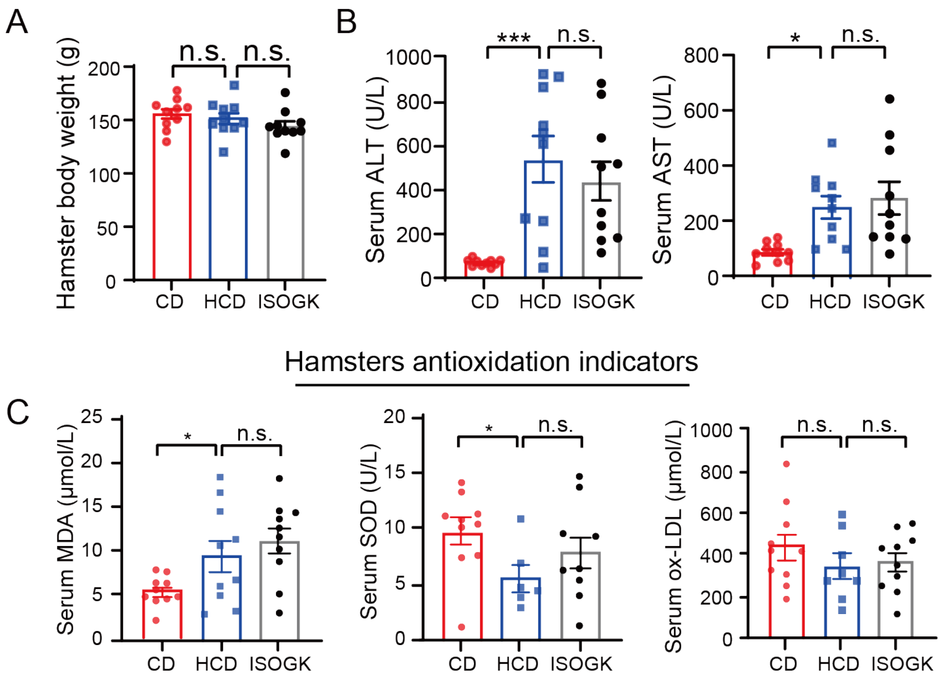

Fig. S4 Effect of ISOGK on body weights, serum ALT, AST and antioxidant parameters in hypercholesterolemic hamsters

(A-B) The body weight and serum ALT, AST levels in the indicated hamster groups. Male hamsters were fed high cholesterol diet for 2 weeks and then received vehicle or ISOGK treatment for an additional 4 weeks (n = 10). One-way ANOVA followed by Bonferroni's post hoc test. (C) Plasma levels of MDA, SOD, ox-LDL in the indicated hamster's group (n = 6 - 10). One-way ANOVA followed by Bonferroni's post hoc test. The data are means ± SEM, n.s., not significant, **P* < 0.05, ****P* < 0.001.

Figure S5

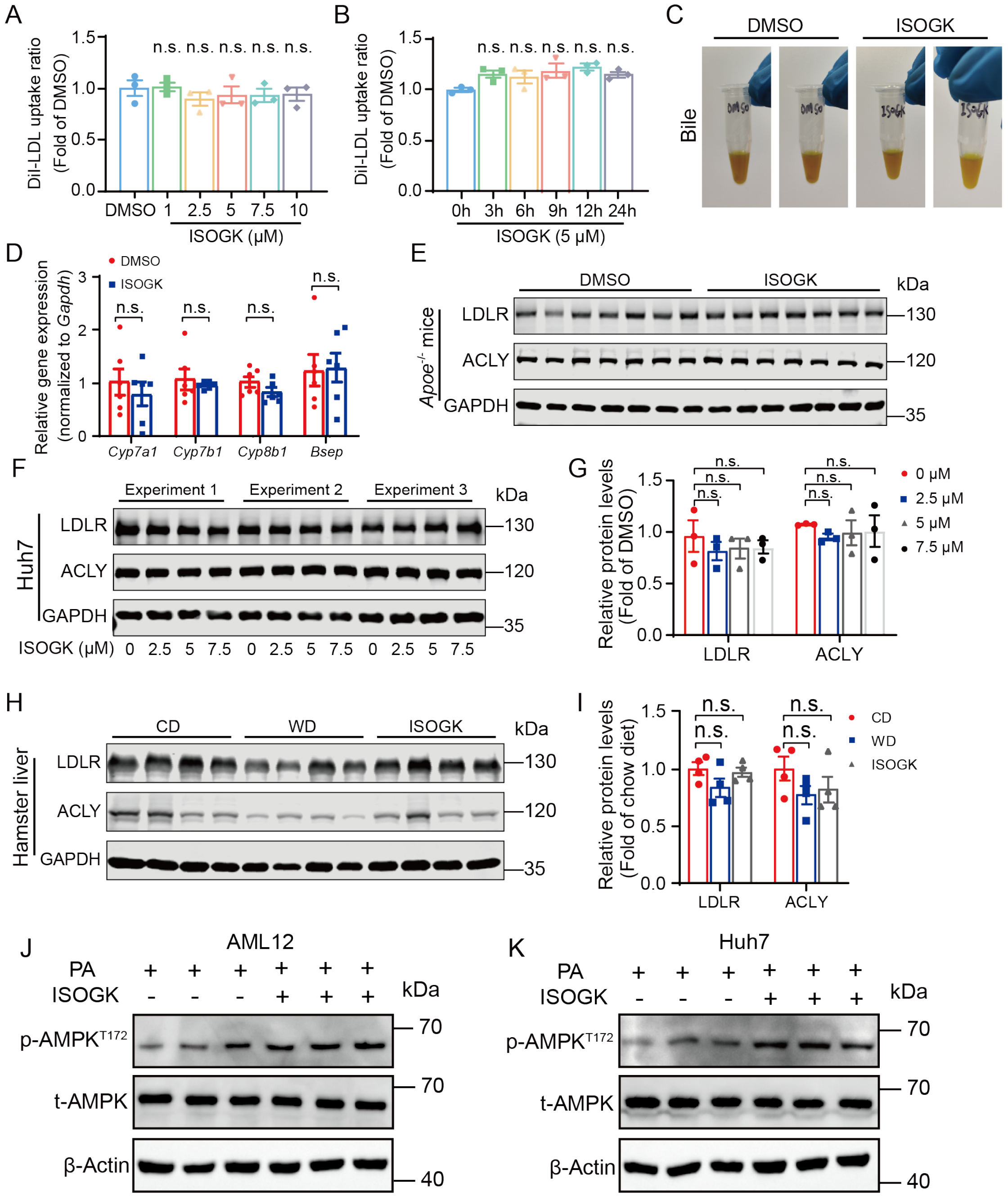

Fig. S5 Isoginkgetin does not affect cholesterol uptake or excretion but activate AMPKα pathway

(A) DiI-LDL uptake ratio in HepG2 cells treated with different doses of ISOGK (n = 3). (B) DiI-LDL uptake ratio in HepG2 cells treated with ISOGK for different times (n = 3). (C) Representative images of the bile from male *Apoe*$^{-/-}$ mice fed high cholesterol diet 7 weeks and administrated with or without ISOGK 8 weeks. (D) qRT-PCR detected

the indicated mRNA levels in liver tissue from indicated male *Apoe*$^{-/-}$ mice (n = 6). Two-tailed Student's t test. (E) Immunoblotting analysis of liver samples in high cholesterol diet-fed male *Apoe*$^{-/-}$ mice after ISOGK treatment (20 mg/kg/day) for 8 weeks (n = 7). (F) Immunoblotting analysis of LDLR and ACLY in Huh7 cells treated with or without ISOGK at different concentrations. (G) Quantitative analysis of F (n = 3). One-way ANOVA followed by Bonferroni's post hoc test. (H) Immunoblotting analysis of liver samples in hamsters after treatment ISOGK (5 mg/kg/day) for 4 weeks. (I) Quantitative analysis of H (n = 4). One-way ANOVA followed by Bonferroni's post hoc test. (J-K) AML12 and HepG2 cell were treated with 50 μM palmitic acid (PA) for 12 h. Then cells treated with or without 5 μM ISOGK for another 12 h. The protein expression of p-AMPK/AMPK were analyzed by immunoblotting (n = 3). The data are means ± SEM, n.s., not significant.

Figure S6

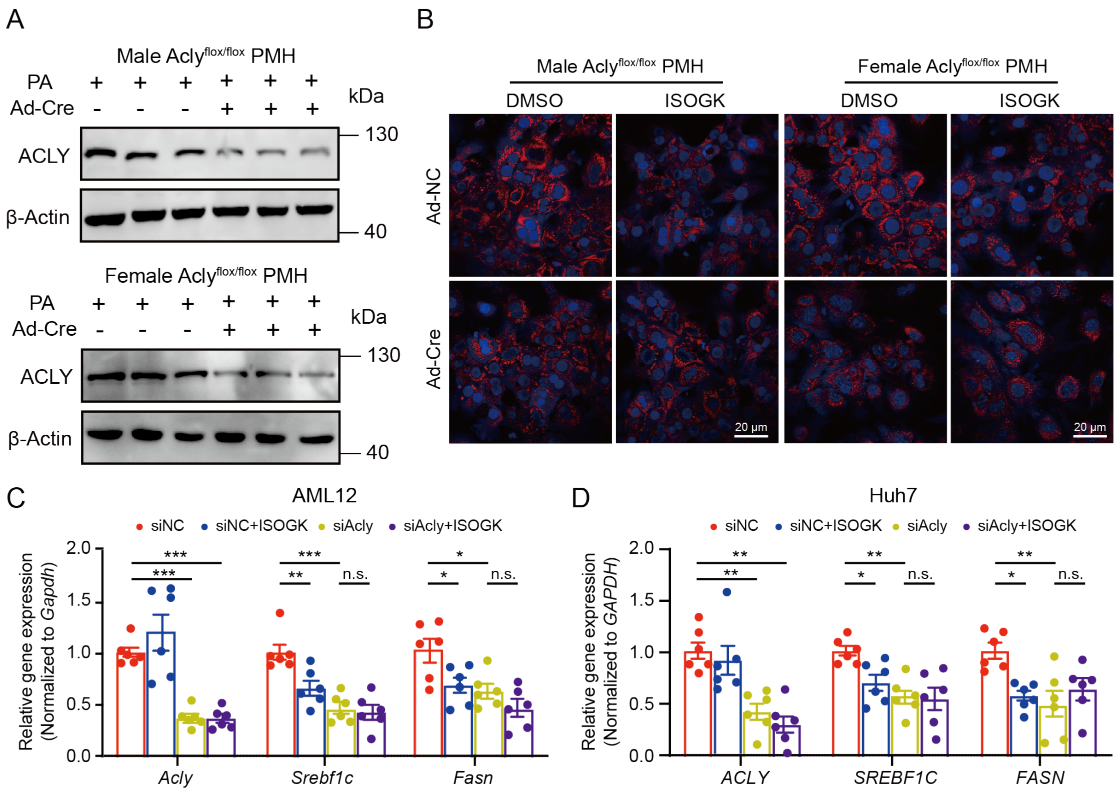

Fig. S6 Knockdown of ACLY reversed the therapeutic effects of ISOGK *in vitro*

(A) Primary mouse hepatocytes (PMH) isolated from one male and one female Acly$^{flox/flox}$ mice were cultured with control or Cre adenovirus for 24 h. Then PMH treated with 50 μM palmitic acid (PA) for another 12 h. The protein expression of ACLY were analyzed by immunoblotting (n=1 per each gender, technical replicates). (B) Representative images of Nile Red staining from PMH treated with or without 5 μM ISOGK for 12 h (n=1 per each gender). (C) qRT-PCR detected the indicated mRNA levels in AML12 cell treated with 50 μM PA and 5 μM ISOGK for 12 h (n=6). One-way ANOVA followed by Bonferroni's post hoc test. (D) qRT-PCR detected the indicated mRNA levels in Huh7 cell treated with 50 μM PA and 5 μM ISOGK for 12 h (n=6). One-way ANOVA followed by Bonferroni's post hoc test.

Figure S7

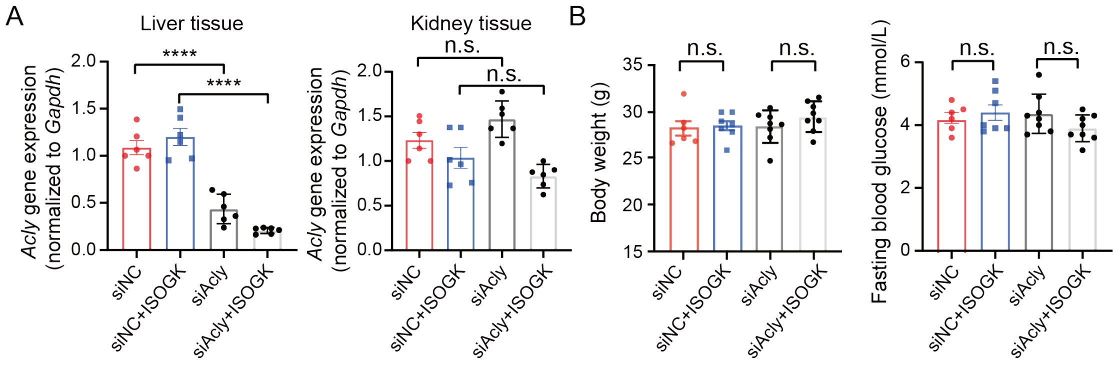

Fig. S7 Effect of ISOGK on body weight and blood glucose in control and Acly-silenced *Apoe$^{-/-}$* mice.

(A) The Acyl mRNA level was detected by qRT-PCR in the liver or kidney tissue from indicated groups of mice (n=6). One-way ANOVA followed by Bonferroni's post hoc test. (B) The body weight and blood glucose levels from male *Apoe$^{-/-}$* mice were induced by high cholesterol diet for 7 weeks and administrated control siRNA or GalNAc-siAcly (3 mg/kg/month) then received ISOGK (20 mg/kg/day) treatment for another 8 weeks (n=6 or 8). One-way ANOVA followed by Bonferroni's post hoc test. The data are means ± SEM, n.s., not significant, ****$P < 0.0001$.

Table S1. Primer sequences for real-time PCR

Primers	Sequences (5’ – 3’)
mGapdh-S	AACAGCAACTCCCACTCTTC
mGapdh-AS	CCTGTTGCTGTAGCCGTATT
mAcly-S	CGGGAGGAAGCTGATGAATATG
mAcly -AS	GTCAAGGTAGTGCCCAATGAA
mCyp7a1-S	ATCACAAACTCCCTGTCATACC
mCyp7a1-AS	CATCACTTGGGTCTATGCTTCT
mCyp7b1-S	CTCGTGAACCACCCTTGATAA
mCyp7b1-AS	GTGTCACCATGTTGCCTTTG
mCyp8b1-S	TTTCTGAGGGAGCAAGGAATAG
mCyp8b1-AS	GGAATAAGAGGACCCAGAAACA
mBsep-S	AACTGAACTTGGAAAGGGGTGT
mBsep-AS	AGCAGAGAAGGCCCTACAGA
mSrebf1c-S	CATCGACTACATCCGCTTCTT
mSrebf1c -AS	CACCAGGTCCTTCAGTGATTT
mFasn-S	AGACCCGAACTCCAAGTTATTC
mFasn-AS	GCAGCTCCTTGTATACTTCTCC
hACLY-S	TGCACTGGAAGTAGAGAAGATTAC
hACLY-AS	AAACTGTGGGTCCTTTACTCG
hSREBF1C-S	CGCTCCTCCATCAATGACAA
hSREBF1C-AS	GTGTTGCAGAAAGCGAATGTAG
hFASN-S	CTAGGTTTGATGCCTCCTTCTT
hFASN-AS	GATGGCTTCATAGGTGACTTCC

S, sense; AS, anti-sense

Table S2. Top 20 candidates in molecular docking study

Rank	Affinity (kcal/mol)	Name	Plant
1	-9.8	Silibinin	*Silybum Adans*
2	-9.7	Fargesin	*Convallaria majalis L.*
3	-9.7	Diosmin	*Amentotaxus argotaenia*
4	-9.6	ISOGINKGETIN	*Ginkgo biloba*
5	-9.6	Rubusoside	*Rubus alceaefolius Poir.*
6	-9.5	Methyl-Hesperidin	*Citrus sinensis*
7	-9.5	Bilobetin	*Ginkgo biloba*
8	-9.5	Sciadopitysin	*Ginkgo biloba*
9	-9.5	Genistin	*Sophora japonica*
10	-9.4	Veratrosine	*Veratrum nigrum L.*
11	-9.4	Linarin	*Uncaria rhynchophylla*
12	-9.4	Ononin	*Ononis arvensis*
13	-9.3	Dihydrosanguinarine	*Chelidonium majus*
14	-9.3	Ginkgetin	*Ginkgo biloba*
15	-9.3	Coreopsin	*Coreopsis basalis*
16	-9.3	Cynaroside	*Cynara scolymus*
17	-9.3	Aurantiamide acetate	*The herbs of Walsura yunnanensis*
18	-9.3	Apigenin 7-glucoside	*Cosmos bipinnatus*
19	-9.3	Glabrone	*The herbs of Cudrania tricuspidata*
20	-9.3	Bilirubin	*Miscellaneous*
